# The Relationship Between Plant-Based Diet and Risk of Digestive System Cancers: A Meta-Analysis Based on 3,059,009 Subjects

**DOI:** 10.3389/fpubh.2022.892153

**Published:** 2022-06-03

**Authors:** Yujie Zhao, Junyi Zhan, Yongsen Wang, Dongli Wang

**Affiliations:** ^1^Department of Gastroenterology, Affiliated Hospital of Shandong University of Traditional Chinese Medicine, Jinan, China; ^2^Graduate School, Shandong University of Traditional Chinese Medicine, Jinan, China

**Keywords:** plant-based diet, meta-analysis, digestive system, eating habits, cancer

## Abstract

**Background and Objectives:**

Diets containing red or processed meat are associated with a growing risk of digestive system cancers. Whether a plant-based diet is protective against cancer needs a high level of statistical evidence.

**Methods:**

We performed a meta-analysis of five English databases, including PubMed, Medline, Embase, Web of Science databases, and Scopus, on October 24, 2021 to identify published papers. Cohort studies or case-control studies that reported a relationship between plant-based diets and cancers of the digestive system were included. Summary effect-size estimates are expressed as Risk ratios (RRs) or Odds ratios (ORs) with 95% confidence intervals and were evaluated using random-effect models. The inconsistency index (I^2^) and τ^2^ (Tau^2^) index were used to quantify the magnitude of heterogeneity derived from the random-effects Mantel-Haenszel model.

**Results:**

The same results were found in cohort (adjusted RR = 0.82, 95% CI: 0.78–0.86, *P* < 0.001, *I*^2^ = 46.4%, Tau^2^ = 0.017) and case-control (adjusted OR = 0.70, 95% CI: 0.64–0.77, *P* < 0.001, *I*^2^ = 83.8%, Tau^2^ = 0.160) studies. The overall analysis concluded that plant-based diets played a protective role in the risk of digestive system neoplasms. Subgroup analyses demonstrated that the plant-based diets reduced the risk of cancers, especially pancreatic (adjusted RR = 0.71, 95% CI: 0.59–0.86, *P* < 0.001, *I*^2^ = 55.1%, Tau^2^ = 0.028), colorectal (adjusted RR = 0.76, 95% CI: 0.69–0.83, *P* < 0.001, *I*^2^ = 53.4%, Tau^2^ = 0.023), rectal (adjusted RR = 0.84, 95% CI: 0.78–0.91, *P* < 0.001, *I*^2^ = 1.6%, Tau^2^ = 0.005) and colon (adjusted RR = 0.88, 95% CI: 0.82–0.95, *P* < 0.001, *I*^2^ = 0.0%, Tau^2^ = 0.000) cancers, in cohort studies. The correlation between vegan and other plant-based diets was compared using Z-tests, and the results showed no difference.

**Conclusions:**

Plant-based diets were protective against cancers of the digestive system, with no significant differences between different types of cancer.

**Systematic Review Registration:**

https://www.crd.york.ac.uk/prospero/display_record.php?ID=CRD42022322276, Identifier: CRD42022322276.

## Introduction

Common digestive system cancers include liver, esophageal, gastric, and colorectal tumors, which are among the 10 most significant healthcare issues worldwide (1). According to the latest statistics in the 2020 GLOBOCAN database, more than 1.9 million new colorectal cancer (including anus) cases and 935,000 deaths occurred (2), and the cancer burden could rise to 27.5 million new cases annually by 2040. Gastric tumors were responsible for over one million new cases and an estimated 769,000 deaths, ranking fifth for incidence and fourth for mortality globally. Liver cancer was the sixth most commonly diagnosed cancer and the third leading cause of cancer death worldwide (906,000 new cases and 830,000 deaths), and esophageal cancer ranked seventh in incidence (604,000 new cases) and sixth in mortality overall (544,000 deaths). Therefore, it is urgent and essential to establish primary prevention programs for digestive system cancers (3). Because 30–50% of all cancer cases are preventable, the World Cancer Research Fund (WCRF) and American Institute for Cancer Research (AICR) published 10 cancer prevention recommendations that set the benchmark for evidence-based guidance, and diet was particularly important (4).

Diet is an inseparable part of people's daily lives, and it has attracted much attention. High quality evidence investigated that red meat, especially the consumption of processed meat, was associated with a growing risk of digestive system cancers (5, 6). Increasing emphasis has been placed on the tumor-preventing function of plant-based diets (7). However, a recent meta-analysis (8) suggested that vegetarian diets were not significantly associated with a lower risk of breast, colorectal or prostate cancer compared to non-vegetarian diets. This study systematically searched two databases and included six cohort studies included with limited types of digestive system cancers. Therefore, the evidence is not sufficiently strong to evaluate the relationship between digestive system cancers and plant-based diets. Comprehensive evaluations are scarce, especially for various digestive system cancers and multiple dietary patterns.

Several styles of vegetarian diets are defined based on the specific animal products consumed (9). Vegetarian diets are classified into six different types according to food selection (10, 11). The vegetarian diets (12) include vegan (eats only plant-based foods but no red meat, poultry, fish, dairy or eggs), pesco-lacto-ovo-vegetarian (eats fish, dairy and eggs without red meat or poultry), lacto-ovo-vegetarian (eats dairy and eggs without red meat, poultry or fish), pesco-vegetarian (eats fish, but no red meat, poultry, dairy or eggs), ovo-vegetarian (eats eggs but no red meat, poultry, fish or dairy), lacto-vegetarian(eats dairy, but no red meat, poultry, fish or eggs) and semi vegetarian (eats dairy, eggs and some red meat, poultry and fish ≥1 time/month but only 1 time/week).

Other classified dietary patterns, such as the Mediterranean diet (13), prudent diet (14) and dietary approaches to stop hypertension (DASH) (15), are widely defined and followed. Because these three diets also focus on vegetables, fruits, and cereals, they were considered plant-based diets. In summary, plant-based diets were defined as follows: (1) a diet excluding any meat, meat products, seafood, or food of animal origin (i.e., vegetarian and vegan diets, respectively); and (2) a diet characterized by a higher consumption of fruits, vegetables, legumes, and nuts rather than animal products (16).

With these complicated classifications, the dietary patterns and subtypes of cancer require further detailed grouping. Therefore, we did this meta-analysis to better assess the association between plant-based diets and gastrointestinal cancers to provide evidence for dietary guidance.

## Methods

### Registration and Reporting Format

The performance of the meta-analysis adhered to the guidelines in the Preferred Reporting Items for Systematic Reviews and Meta-analyses (PRISMA) statement (17) and Meta-analysis of Observational Studies in Epidemiology (MOOSE) statement (18). The PRISMA and MOOSE checklists are presented in Supplementary Tables 1, 2. The study protocol was registered with the International Prospective Register of Systematic Reviews (PROSPERO), and the registration number is CRD42022322276.

### Search Strategy

A literature search was performed in the PubMed, Medline, Embase, Web of Science and Scopus databases before October 24, 2021. The PICOS tool was used to guide the search strategy: (P) Population: patient with digestive system cancers; (I) Intervention: plant-based diet; (C) Comparator: other diet patterns; (O) Outcomes: gastrointestinal system cancers; (S) Study type: case-control and cohort studies. A complete list of the search terms is available in the additional materials section (Supplementary Table 3).

### Eligibility Criteria

Our analysis was restricted to articles that met the following criteria: (1) study participants: population with plant-based diets; (2) endpoints: all kinds of digestive system cancers; (3) study type: cohort studies or case-control studies; (4) follow-up rate: at least 70%; and (5) the dietary patterns in the articles included specific food components. Case reports or case series, editorials, narrative reviews, non-English articles,and literature with unqualified data and not available were excluded.

### Study Selection

Endnote 20 literature management software was used to manage the literature search records. The selection process covered three sections. Two reviewers (Y.Z. and Y.W.) independently reviewed articles based on their titles, and duplicates were removed. Articles with questionable titles were included in the abstract review phase. The same two independent reviewers screened and evaluated the abstracts of all articles selected from the first section for eligibility. Meanwhile, they assessed full-text articles that warranted further investigation using the eligibility criteria. Disagreements in each phase were resolved by a third independent reviewer (D.W.) from our group.

### Data Extraction

Two investigators (Y.Z. and Y.W.) independently extracted data from each qualified article, including the first author, year of publication, country where study was performed, sex, sample size, study type, follow-up years, the age of study subjects, cancer type, definition of the vegetarian dietary pattern, menstrual status and other confounding risk factors, when available. The divergence was resolved via joint re-evaluations of original articles, and by a third author (D.W.) when necessary.

### Risk of Bias of Individual Studies

Two authors (Y.Z. and Y.W.) independently assessed the risk of bias of all eligible studies using the Risk of Bias in Non-randomized Studies-of Interventions (ROBINS-I) assessment tool (19). The following seven domains were considered: (i) bias due to confounding; (ii) bias in selection of participants into the study; (iii) bias in classification of interventions; (iv) bias due to deviations from intended interventions; (v) bias due to missing data; (vi) bias in measurement of outcomes; (vii) bias in selection of the reported result. Each part were categorized into five levels of ROB: low risk of bias (the study is comparable to a well-performed randomized trial with regard to this domain); moderate risk of bias (the study is sound for a non-randomized study with regard to this domain but cannot be considered comparable to a well- performed randomized trial); serious risk of bias (the study has some important problems); critical risk of bias (the study is too problematic to provide any useful evidence on the effects of intervention); no information (no information is reported about the methods of outcome assessment). Trials were divided into three levels of ROB by the number of components for which high ROB potentially existed: high risk (five or more), moderate risk (three or four) and low risk (two or less).

### Statistical Analyses

Data management and analyses were handled using STATA software version 14.1 for Windows (Stata Corp, College Station, TX, USA). Effect size was estimated as risk ratios (RRs) or hazard ratios (HRs) with 95% confidence intervals (CIs) in cohort studies and as odds ratios (ORs) with 95% CIs in case-control studies. To make the statistical results more accurate, we transformed the HRs into RRs using the formula RR = (1–expHR^*^ln (1–r))/r. Pooled effect-size estimates were derived under the random-effects model, regardless of the magnitude of between-study heterogeneity. Differences between two estimates were tested using the Z-test proposed by Altman and Bland (20).

The inconsistency index (I^2^) statistic, which represents the percent of diversity due to heterogeneity rather than chance, was used to quantify the magnitude of heterogeneity derived from the random-effects Mantel-Haenszel model. An *I*^2^ > 50% indicated the presence of significant heterogeneity, and a higher percent corresponded to a higher degree of heterogeneity. Another index, τ^2^ (Tau^2^), was used to examine the sensitivity of the results to different levels of between-study heterogeneity. To account for possible sources of between-study heterogeneity from clinical and methodological aspects, a large number of pre-specified subgroup analyses were performed according to geographic region, study design, age, sex, cancer type, definition of plant-based dietary pattern, and follow-up interval. To avoid giving large weight to relatively small studies, we fitted the fixed effect models using sensitivity analyses.

The probability of publication bias was evaluated using Begg's funnel plots and Egger regression asymmetry tests at a significance level of 10%. The trim-and-fill method was used to estimate the number of theoretically missing studies.

## Results

### Eligible Studies

**Figure 1:**
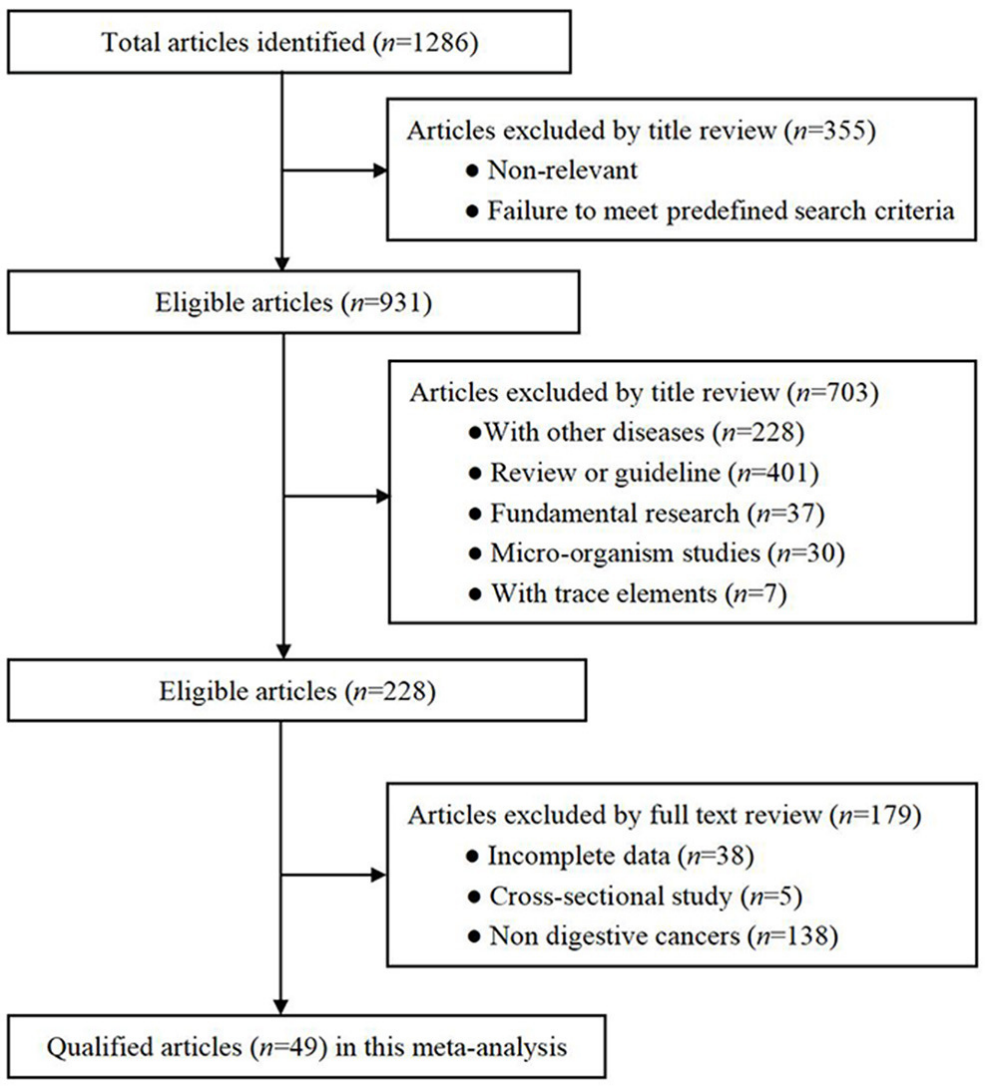
Flow chart of records retrieved, screened and included in this meta-analysis.

A total of 5,232 articles were initially identified using predefined medical subject words to search the predefined public database, and 49 of studies met the criteria for eventual inclusion, including 3,059,009 subjects. The detailed selection process is shown in Figure 1.

### Study Characteristics

Supplementary Table 4 shows the baseline characteristics of the 49 articles (21–69) included in this meta-analysis. Eighteen of these articles were cohort studies (21, 24–27, 32, 33, 36, 40, 41, 47, 51, 55, 57, 59, 61, 62, 69), and 31 were case-control studies (22, 23, 28–31, 34, 35, 37–39, 42–46, 48–50, 52–54, 56–58, 60, 63–68). Sixteen articles were performed in Europe (22, 24, 29, 33, 37, 40, 42, 43, 45, 48–50, 52, 59–61), 15 articles were attributed to North America (21, 23, 26, 28, 30, 32, 35, 36, 38, 41, 44, 54, 56, 57, 68), 16 articles were Asian (25, 27, 31, 34, 46, 47, 51, 53, 58, 62–67, 69) and the remaining 2 articles were from Oceania (39, 55). Depending on the study endpoints, 6 articles focused on pancreatic cancer (21, 28, 41–44), 23 articles focused on colorectal cancer (22, 24, 27, 29, 34–37, 40, 45, 46, 50, 51, 53–56, 58, 59, 61–64), 12 studies examined colon cancer (23, 24, 26, 27, 37, 51, 55–57, 59, 61, 62), 12 studies were performed on rectal cancer (24, 27, 32, 37, 51, 55–59, 61, 62), 9 studies investigated gastric cancer (25, 30, 33, 48, 52, 60, 65–67), 3 studies focused on esophageal cancer (31, 39, 69), 2 studies examined pharyngolaryngeal cancer (38, 49), and 2 articles were on liver cancer (47, 68). Fifteen of the included articles used female groups (24, 27, 28, 30, 32, 36, 40, 41, 44, 47, 56, 58–60, 66) and 16 studies examined male subjects (25–28, 30, 32, 35, 36, 40, 41, 44, 47, 56, 58, 60, 66). We created a detailed classification of the dietary patterns involved according to the principles mentioned in the incorporated eligible article (Supplementary Table 5). Classification resulted in 14 articles on vegan diets (21, 22, 25, 28, 29, 31, 35, 36, 39, 43, 47, 51, 54, 57), 8 articles contained semi-vegetarian diets (24, 32, 37, 38, 41, 48, 61, 63), 1 article was on pesco-vegetarians (27), 1 study mentioned lacto-ovo-vegetarians (55), 2 studies reported on pesco-laco-ovo-vegetarians (46, 53), 1 study involved lacto-vegetarians (68), 10 articles were on prudent diets (23, 26, 27, 30, 34, 44, 56, 58, 60, 62), 3 studies referred to the DASH diet (56, 65, 66) and 11 articles were on the Mediterranean diet (33, 40, 42, 45, 49, 50, 52, 59, 60, 64, 69).

### Results of ROB Assessment

The details of the ROB assessment in each study we included was showed in Supplementary Table 6. Overall, 32 articles were judged to be of low ROB, 14 articles were rated to be of moderate risk and 2 were of high risk. In the box of confounding bias, 31 articles showed low ROB and 2 were rated as serious. In the box of selection bias, 16 studies were judged to be of low risk and the remaining 32 were of moderate risk. For the bias of missing data, 25 articles presented the low ROB and for bias due to deviations from intended interventions, 24 studies didn't show the detail information.

### Overall Analyses

After summarizing the results of all qualified cohort and case-control studies, the pattern of plant-based diets was statistically associated with the risk of digestive system cancer. The results suggested that a plant-based diet pattern played a protective factor for the risk of digestive system cancer in the cohort studies (adjusted RR = 0.82, 95% CI: 0.78–0.86, *P* < 0.001, *I*^2^ = 46.4%, Tau^2^ = 0.017) and case-control studies (adjusted OR = 0.70, 95% CI: 0.64–0.77, *P* < 0.001, *I*^2^ = 83.8%, Tau^2^ = 0.160).

**Figure 2:**
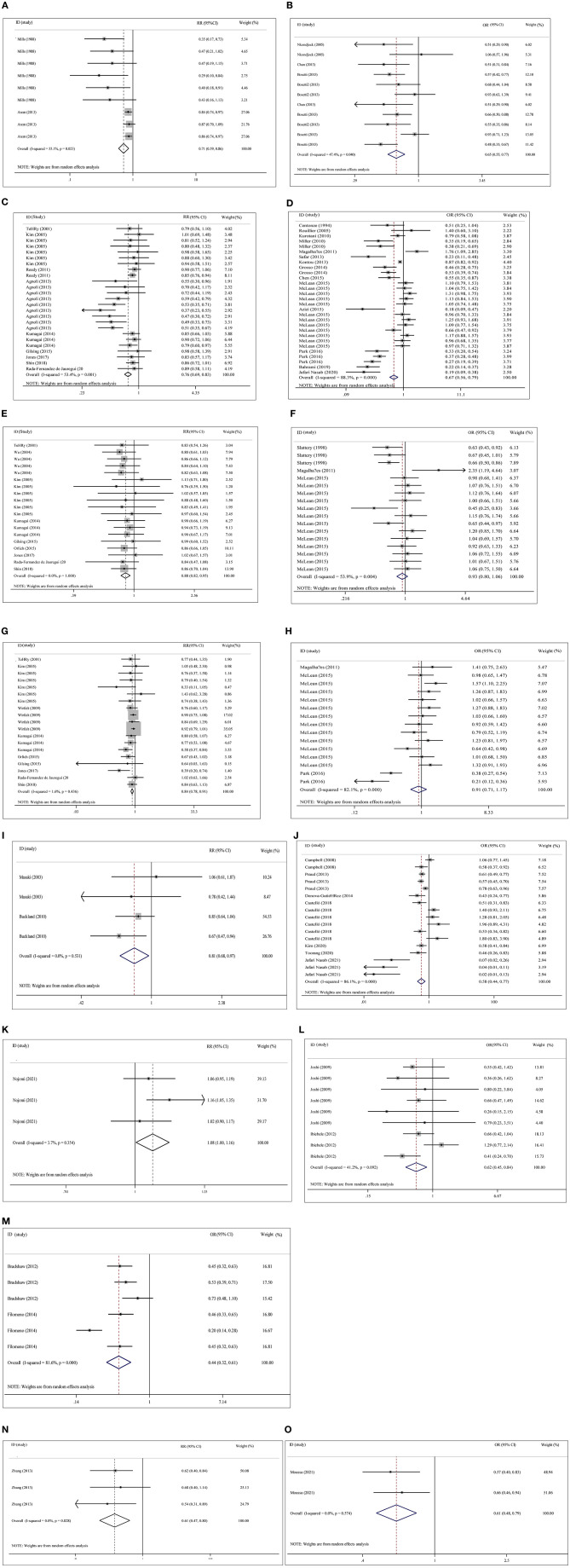
Overall analysis of plant-based diets and cancer risk in cohort studies and case-control studies with risk ratio (RR), odds ratio (OR) and 95% confidence interval (CI). **(A)** Pancreas cancer with cohort study in overall analysis (adjusted). **(B)** Pancreas cancer with case- control study in overall analysis (adjusted). **(C)** Colorectal cancer with cohort study in overall analysis (adjusted). **(D)** Colorectal cancer with case-control study in overall analysis (adjusted). **(E)** Colon cancer with cohort study in overall analysis (adjusted). **(F)** Colon cancer with case- control study in overall analysis (adjusted). **(G)** Rectal cancer with cohort study in overall analysis (adjusted). **(H)** Rectal cancer with case-control study in overall analysis (adjusted). **(I)** Gastric cancer with cohort study in overall analysis (adjusted). **(J)** Gastric cancer with case-control study in overall analysis (adjusted). **(K)** esophagus cancer with cohort study in overall analysis (adjusted). **(L)** esophagus cancer with case- control study in overall analysis (adjusted). **(M)** Pharyngolaryngeal cancer with case-control study in overall analysis (adjusted). **(N)** Liver cancer with cohort study in overall analysis (adjusted). **(O)** Liver cancer with case- control study in overall analysis (adjusted).

Plant-based diets were statistically significant for pancreas cancer (adjusted RR = 0.71, 95% CI: 0.59–0.86, *P* < 0.001, *I*^2^ = 55.1%, Tau^2^ = 0.028), colorectal cancer (adjusted RR = 0.76, 95% CI: 0.69–0.83, *P* < 0.001, *I*^2^ = 53.4%, Tau^2^ = 0.023), colon cancer (adjusted RR = 0.88, 95% CI: 0.82–0.95, *P* < 0.001, *I*^2^ = 0.0%, Tau^2^ = 0.000), rectal cancer (adjusted RR = 0.84, 95% CI: 0.78–0.91, *P* < 0.001, *I*^2^ = 1.6%, Tau^2^ = 0.005), gastric cancer (adjusted RR = 0.81, 95% CI: 0.68–0.97, *P* = 0.021, *I*^2^ = 0.0%, Tau^2^ = 0.000), liver cancer (adjusted RR = 0.61, 95% CI: 0.47–0.80, *P* < 0.001, *I*^2^ = 0.0%, Tau^2^ = 0.000), and esophageal cancer (adjusted RR = 1.08, 95% CI: 1.00–1.16, *P* = 0.04, *I*^2^ = 3.7%, Tau^2^ = 0.001) in the cohort studies, and equivalent connections were found in case-control studies for pancreatic cancer (adjusted OR = 0.65, 95% CI: 0.55–0.77, *P* < 0.001, *I*^2^ = 47.4%, Tau^2^ = 0.035), colorectal cancer (adjusted OR = 0.67, 95% CI: 0.56–0.80, *P* < 0.001, *I*^2^ = 88.3%, Tau^2^ = 0.177), gastric cancer (adjusted OR = 0.58, 95% CI: 0.44–0.77, *P* < 0.001, *I*^2^ = 86.1%, Tau^2^ = 0.252), pharyngolaryngeal cancer (adjusted OR = 0.44, 95% CI: 0.32–0.61, *P* < 0.001, *I*^2^ = 81.6%, Tau^2^ = 0.135) and liver cancer (adjusted OR = 0.61, 95% CI: 0.48–0.79, *P* < 0.001, *I*^2^ = 0.0%, Tau^2^ = 0.000). No statistically significant relationship was found between plant-based diets and colon cancer (adjusted OR = 0.93, 95% CI: 0.80–1.06, *P* = 0.280, *I*^2^ = 53.9%, Tau^2^ = 0.045) or rectal cancer (adjusted OR = 0.91, 95% CI: 0.71–1.17, *P* = 0.469, *I*^2^ = 82.1%, Tau^2^ = 0.204) (Figure 2).

### Cumulative and Sensitivity Analyses

Cumulative analysis of the included studies obtained completely similar conclusions, and the trend tended to be stable. Sensitivity analyses revealed no significant impact on any single study on overall effect-size estimates.

### Publication Bias

**Figure 3:**
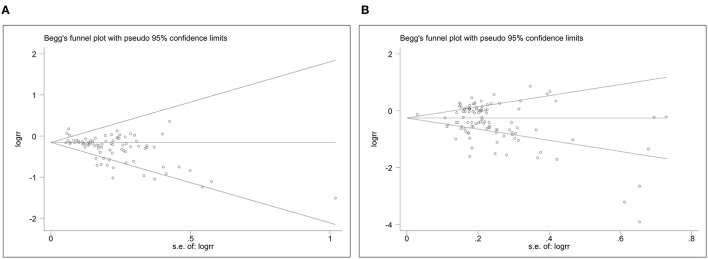
The Begg's funnel plots for plant-based diets and cancer risk. **(A)**, Plant-based diets and cancer risk in cohort studies. **(B)**, Plant-based diets and cancer risk in case-control studies.

Figure 3 shows Begg's funnel plot of publication bias for the association of plant-based diets with digestive system cancers. Evidence of asymmetry of study effects was found using Eggers's test in the cohort (Coef. = −1.43, 95%CI: −1.94 to −0.93, *P* = 0.000) and case-control studies (Coef. = −1.23, 95%CI: −2.08 to −0.38, *P* = 0.005). The “trim and fill” method was used and no correction was made to the original estimates.

### Subgroup Analyses

Because of between-study heterogeneity, we performed a series of pre-specified analyses to further explore the relationship between plant-based diets and digestive system cancer risk. Notably, the protective effect of plant-based diet patterns was accordant in all subgroup analyses (Table 1). We did not find a consistent pattern of difference or heterogeneity in the results by sex, or any other study characteristics examined in the selected cohort studies. However, significant heterogeneity was found in the results of the included case-control studies, including sex, geographic region, type of digestive cancers, classification of plant-based diets, and follow-up intervals.

**Table 1:** Overall and subgroup analyses of plant-based diets and digestive cancer risk.

**Groups**	**Number of qualified observations**	**Plant-based diets (cohort study)**			**Plant-based diets (case-control study)**		
		**RR (95% CI); *P***	***I^**2**^***	***Tau^**2**^***	**OR (95% CI); *P***	***I* ^2^**	***Tau^**2**^***
**Overall analyses**							
Digestive system cancer (adjusted)	82/106	0.82 (0.78–0.86); <0.001	46.4%	0.017	0.70 (0.64–0.77); <0.001	83.8%	0.160
**Subgroup analyses based on Cancer**							
**By cancer type**							
Pancreas cancer	9/11	0.71 (0.59–0.86); <0.001	55.1%	0.028	0.65 (0.55–0.77); <0.001	47.4%	0.035
Colorectal cancer	25/29	0.76 (0.69–0.83); <0.001	53.4%	0.023	0.67 (0.56–0.80); <0.001	88.3%	0.177
Colon cancer	19/17	0.88 (0.82–0.95); 0.001	0.0%	0.000	0.93 (0.80–1.06); 0.277	53.9%	0.045
Rectal cancer	19/15	0.84 (0.78–0.91); <0.001	1.6%	0.005	0.91 (0.71–1.17); 0.469	82.1%	0.204
Gastric cancer	4/17	0.81 (0.68–0.97); 0.021	0.0%	0.000	0.58 (0.44–0.77); <0.001	86.1%	0.252
Esophagus cancer	3/9	1.08 (1.00–1.16); 0.040	3.7%	0.001	0.62 (0.45–0.84); 0.002	41.2%	0.084
Pharyngolaryngeal cancer	*/6	*	*	*	0.44 (0.32–0.61); <0.001	81.6%	0.135
Liver cancer	3/2	0.61 (0.47–0.80); <0.001	0.0%	0.000	0.61 (0.48–0.79); <0.001	0.0%	0.000
**By sex**							
Male	23/28	0.85 (0.81–0.90); <0.001	46.4%	0.017	0.79 (0.65–0.95); 0.015	85.6%	0.223
Female	23/26	0.77 (0.69–0.87); <0.001	45.8%	0.031	0.92 (0.81–1.05); 0.236	58.9%	0.068
Both	34/49	0.81 (0.75–0.88); <0.001	63.7%	0.029	0.57 (0.50–0.65); <0.001	85.5%	0.161
**By region**							
North America	21/54	0.84 (0.80–0.89); <0.001	13.4%	0.002	0.88 (0.80–0.96); 0.004	65.7%	0.070
Europe	20/28	0.67 (0.59–0.76); <0.001	49.8%	0.039	0.72 (0.61–0.84); <0.001	86.0%	0.135
Asia	38/21	0.90 (0.85–0.96); 0.001	24.1%	0.007	0.30 (0.23–0.40); <0.001	79.3%	0.281
Oceania	3/3	0.97 (0.71–1.33); 0.871	0.0%	0.000	0.71 (0.38–1.32); 0.274	78.7%	0.239
**By population**							
Adventist	8/*	0.54 (0.39–0.74); <0.001	48.4%	0.085	*	*	*
Normal person	74/106	0.83 (0.79–0.87); <0.001	43.9%	0.015	0.70 (0.64–0.77); <0.001	83.8%	0.160
**By plant-based diets**							
Vegan	24/19	0.80 (0.74–0.86); <0.001	27.2%	0.008	0.62 (0.52–0.75); <0.001	43.2%	0.067
Semi vegetarian	13/8	0.87 (0.82–0.92); <0.001	0.0%	0.000	0.73 (0.45–1.18); 0.195	89.0%	0.421
Lacto-vegetarian	*/2	*	*	*	0.61 (0.48–0.79); <0.001	0.0%	0.000
Pesco-vegetarian	18/*	0.90 (0.80–1.03); 0.118	0.0%	0.000	*	*	*
Lacto-ovo-vegetarian	3/*	0.97 (0.71–1.33); 0.871	0.0%	0.000	*	*	*
Pesco-laco-ovo-vegetarian	*/2	*	*	*	0.21 (0.12–0.36); <0.001	0.0%	0.000
DASH diets	*/40	*	*	*	0.96 (0.86–1.08); 0.499	68.9%	0.082
Prudent diets	7/18	0.85 (0.77–0.92); <0.001	0.0%	0.000	0.59 (0.46–0.75); <0.001	84.6%	0.220
Mediterranean diet	17/17	0.69 (0.59–0.82); <0.001	84.3%	0.091	0.55 (0.46–0.67); <0.001	89.5%	0.136
Non-vegan	58/87	0.82 (0.77–0.87); <0.001	56.2%	0.026	0.72 (0.65–0.79); <0.001	85.8%	0.165
**By follow-up years**							
≤ 10 years	39/*	0.83 (0.78–0.88); <0.001	31.4%	0.008	*	*	*
>10 years	40/*	0.79 (0.73–0.86); <0.001	49.9%	0.021	*	*	*

RR, risk ratio; OR, odds ratio; 95% CI, 95% confidence interval; DASH, Dietary Approaches to Stop Hypertension; *, data not available.

Plant-based diets were statistically associated with digestive system cancer risk in males (adjusted RR = 0.85, 95% CI: 0.81–0.90, *P* < 0.001, *I*^2^ = 46.4%, Tau^2^ = 0.017) and females (adjusted RR = 0.77, 95% CI: 0.69–0.87, *P* < 0.001, *I*^2^ = 45.8%, Tau^2^ = 0.031) in the cohort studies (two-sample Z-test *P* = 0.128).

The included cohort and case-control studies were divided into North America, Europe, and Asia. In the cohort studies, subgroup analysis demonstrated statistical significance of plant-based dietary patterns for digestive cancers in Europe (adjusted RR = 0.67, 95% CI: 0.59–0.76, *P* < 0.001, *I*^2^ = 49.8%, Tau^2^ = 0.039), Asia (adjusted RR = 0.90, 95% CI: 0.85–0.96, *P* = 0.001, *I*^2^ = 24.1%, Tau^2^ = 0.007) or North America (adjusted RR = 0.84, 95% CI: 0.80–0.89, *P* < 0.001, *I*^2^ = 13.4%, Tau^2^ = 0.002). In the case-control studies, subgroup analysis proved statistical significance of plant-based dietary patterns for digestive cancers in Europe (adjusted OR = 0.72, 95% CI: 0.61–0.84, *P* < 0.001, *I*^2^ = 86.0%, Tau^2^ = 0.135), Asia (adjusted OR = 0.30, 95% CI: 0.23–0.40, *P* < 0.001, *I*^2^ = 79.3%, Tau^2^ = 0.281) or North America (adjusted OR = 0.88, 95% CI: 0.80–0.96, *P* = 0.004, *I*^2^ = 65.7%, Tau^2^ = 0.070).

We found a significant difference between the vegan pattern (adjusted RR = 0.80, 95% CI: 0.74–0.86, *P* < 0.001, *I*^2^ = 27.2%, Tau^2^ = 0.008) and digestive system cancers in the cohort studies, and this relationship also existed in the prudent dietary pattern (adjusted RR = 0.85, 95% CI: 0.77–0.92, *P* < 0.001, *I*^2^ = 0.0%, Tau^2^ = 0.000), semi vegetarian pattern (adjusted RR = 0.87, 95% CI: 0.82–0.92, *P* < 0.001, *I*^2^ = 0.0%, Tau^2^ = 0.000), and Mediterranean pattern (adjusted RR = 0.69, 95% CI: 0.59–0.82, *P* < 0.001, *I*^2^ = 84.3%, Tau^2^ = 0.091). However, there were no significant difference between pesco-vegetarians (adjusted RR = 0.90, 95% CI: 0.80–1.03, P =0.118, *I*^2^ = 0.0%, Tau^2^ = 0.000) and lacto-ovo-vegetarians (adjusted RR = 0.97, 95% CI: 0.71–1.33, P =0.871, *I*^2^ = 0.0%, Tau^2^ = 0.000). Vegan (adjusted OR = 0.62, 95% CI: 0.52–0.75, *P* < 0.001, *I*^2^ = 43.2%, Tau^2^ = 0.067), prudent (adjusted OR = 0.59, 95% CI: 0.46–0.75, *P* < 0.001, *I*^2^ = 84.6%, Tau^2^ = 0.220), Mediterranean (adjusted OR = 0.55, 95% CI: 0.46–0.67, *P* < 0.001, *I*^2^ = 89.5%, Tau^2^ = 0.136), pesco-laco-ovo-vegetarian (adjusted OR = 0.21, 95% CI: 0.12–0.36, *P* < 0.001, *I*^2^ = 0.0%, Tau^2^ = 0.000) and lacto-vegetarian (adjusted OR = 0.61, 95% CI: 0.48–0.79, *P* < 0.001, *I*^2^ = 0.0%, Tau^2^ = 0.000) diets had robust correlations with digestive tract cancers in case-control studies, but not semi-vegetarian (adjusted OR = 0.73, 95% CI: 0.45–1.18, *P* = 0.195, *I*^2^ = 89.0%, Tau^2^ = 0.421) or DASH diets (adjusted OR = 0.96, 95% CI: 0.86–1.08, *P* = 0.499, *I*^2^ = 68.9%, Tau^2^ = 0.082).

We combined plant-based diets other than the vegan pattern into the non-vegan diet and found that vegan and non-vegan diets were statistically significant for digestive cancers, but no significant difference was found between the two diets in cohort studies (two-sample *Z*-test *P* = 0.617) or case-control studies (two-sample *Z*-test *P* = 0.158).

Prominent differences were found in people of the Adventists faith (adjusted RR = 0.54, 95% CI: 0.39–0.74, *P* < 0.001, *I*^2^ = 48.4%, Tau^2^ = 0.085) and normal populations (adjusted RR = 0.83, 95% CI: 0.79–0.87, *P* < 0.001, *I*^2^ = 43.9%, Tau^2^ = 0.015) in cohort studies.

For the median value (10 years) of the follow-up period, the protective effect of plant-based diets for digestive cancers was consistent and significant regardless of the length of follow-up in cohort studies (≤ 10 years: adjusted RR = 0.83, 95% CI: 0.78–0.88, *P* < 0.001, *I*^2^ = 31.4%, Tau^2^ = 0.008; >10 years: adjusted RR = 0.79, 95% CI: 0.73–0.86, *P* < 0.001, *I*^2^ = 49.9%, Tau^2^ = 0.021).

## Discussion

To the best of our knowledge, this study is the first comprehensive examination of meta-analyses between plant-based diets and digestive system tumors. Our key findings suggest protective effects of a plant-based diet on digestive cancer risk in cohort and case-control studies. Our adjunctive analysis showed that geographic region, type of digestive cancer, classification of plant-based diets, and follow-up intervals may be sources of inter-study heterogeneity. The implication of this study is a call for action to pay special attention to plant-based diets to reduce the risk of digestive system cancers.

Our findings are biologically plausible. Inflammation, oxidative stress, and the mediating effect of insulin all affect the development of tumors (70). Oxidative stress causes DNA damage and the risk of cancer if not repaired (71). The process by which insulin and insulin-like growth factors regulate carbohydrate and energy metabolism is associated with cancer risk (72). Inflammation is also a recognized marker of cancer that affects the development and progression of malignant tumors (73).

Plant foods (e.g., fruits, vegetables, grains, nuts and seeds, legumes and vegetable oils) are primary sources of fiber and other bioactive compounds in the diet (16, 74). A well-planned plant-based diet promotes a high intake of vitamins, minerals, and phytochemicals, which regulate antioxidant and anti-inflammatory prcoesses (9, 75). Notably, plant bioactive substances, including fiber, sulfur compounds, carotenoids, and polyphenols, are rich in foods such as cruciferous vegetables, allium vegetables, tomatoes, green tea, and whole grain grains (74), which are beneficial against cancer. Carotenoids promote good health due to their special physiological efficacy as provitamins and antioxidant reactions, especially in the clearance of singlet oxygen, which reduce the risk of cancers (76). Vitamin C, vitamin E, and other natural antioxidants of plants, such as polyphenols, alfalfa, anthocyanins, flavonoids, lignans, and phenolic acids, have a variety of biological properties, such as anti-inflammatory and anti-cancer properties (77–79). More interestingly, Afshari et al. (80) evaluated the anti-cancer properties of eggplant extract on human gastric cancer cell lines. They concluded that eggplant was rich in phenolic components and had powerful antioxidant properties that effectively scavenged free radicals. Therefore, eggplant may be a protective food to reduce the incidence of cancer (81).

Evidences suggests that the effect of a plant-based diet on intestinal flora is inextricably linked to digestive tract tumors. Daily consumption of nuts reduces the risk of cancers of the digestive system. High levels of dietary fiber, polyphenols and unsaturated fats are rich nutrients in nuts, and dietary fiber increases anaerobic fermentation and reduces intestinal transit time, which may reduce the exposure of colorectal mucosa to carcinogens (82). Polyphenols and unsaturated fats increase the abundance of Bifidobacterium and Lactobacillus in the intestine, which inhibit gastrointestinal inflammation and carcinogenic effects by promoting the production of short-chain fatty acids (83). The present study found that vegetarian diets were more protective in Asians. With fast economic growth and rapid industrialization, the thriving middle class in developing countries is adopting a Westernized lifestyle that is characterized by a high-fat, high-protein diet, which may change the community of microbes living in the humans to increase the risk of cancer (84, 85). Notably, the latest data show that East Asia was the worst-affected region, with 637,096 new cases and 275,604 deaths due to colorectal cancer (85).

Due to the protective effect of vegetarian dietary patterns on tumors, we examined whether people needed to ensure a pure vegan diet. For further research, we divided the plant-based dietary patterns into two categories, including a pure vegan diet and other types of primarily vegetarian diets and found that these two diet types produced equivalent protective roles against digestive system cancers. This conclusion means people do not need to adopt a pure plant-based diet. These results provide a more robust understanding of healthy eating guidance. According to WCRF dietary recommendations, people do not need to completely avoid eating meat but should limit consumption to no more than approximately three portions per week. People should consume a diet that provides at least 30 g per day of fiber and five portions or servings (at least 400 g or 15 oz in total) of a variety of non-starchy vegetables and fruit every day (4). These results are consistent with the pant-based diet advocated in the present study.

Our review was systematic and exhaustive and concluded the most different types of digestive cancers and various plant-based diets. A considerable sample size of 3,059,009 subjects and adults living with digestive cancers *(n* = 34,009) were included, which provided the power to detect a statistically significant relationship between plant-based diets and digestive cancers. However, the possible limitations of our meta-analysis must be considered. First, the present meta-analysis involved sufficient sample sizes for overall analyses, but the number of qualified studies in some subgroups was very limited. For example, the number of original articles involving pesco-vegetarian, lacto-vegetarian, and lacto-ovo-vegetarian diets was too small, which results in bias in the results to some extent. Second, although all studies used validated questionnaires to collect dietary data, most studies did not provide repeated measurements during the follow-up periods and did not register possible change in diet over time. Third, several of the analyses involved comparing the highest vs. lowest exposure categories, which may exaggerate associations by focusing on the extremes of the distribution. However, with the relative paucity of studies referring to different exposure levels of plant-based diets, we were not able to perform a dose-response analysis to obtain more detailed guideline results. Although all the selected original articles were detailed in their investigation of food, they differentiated between meat from common poultry and red meat and foods with higher fat content and assessed the definition of plant-based diets using specialized scales. However, we cannot completely exclude the consumption of a mixture of red meat and other meats. The World Health Organization classified processed meat as a Class 1 carcinogen and red meat as a Class 2A carcinogen (86). However, there is no evidence that natural poultry meats have a significant effect on digestive cancers. Finally, the food industry provides a wide variety of vegan foods, which are classified as ultra-processed food due to the degree of processing. Whether vegans are harmed and have an increased risk for digestive system cancers is not clear because they may consume these foods more than non-vegan people. This aspect should be investigated in future studies.

In summary, it is important to understand and reveal eating habits that make our lives healthier and the important role these habits play in the management and prevention of oncological diseases. Our study propose that a plant-based diet is promising to prevent the development of cancer.

## Data Availability Statement

The original contributions presented in the study are included in the article/Supplementary Material, further inquiries can be directed to the corresponding author.

## Author Contributions

DW and YZ: conceived and designed the experiments. YZ and JZ: performed the experiments and wrote the paper. JZ and YW: analyzed the data. YW: contributed materials and analysis tools. All authors read and approved the final manuscript prior to submission.

## Funding

Taishan Scholar Foundation of Shandong Province (Award number(s): 2018–35).

## Conflict of Interest

The authors declare that the research was conducted in the absence of any commercial or financial relationships that could be construed as a potential conflict of interest.

## Publisher's Note

All claims expressed in this article are solely those of the authors and do not necessarily represent those of their affiliated organizations, or those of the publisher, the editors and the reviewers. Any product that may be evaluated in this article, or claim that may be made by its manufacturer, is not guaranteed or endorsed by the publisher.
