# Supplementary material for: The Relationship Between Plant-Based Diet and Risk of Digestive System Cancers: A Meta-Analysis Based on 3,059,009 Subjects
Source: Front Public Health. 2022 Jun 3;10:892153. doi: 10.3389/fpubh.2022.892153 (PMC9204183; doi:10.3389/fpubh.2022.892153)
Supplement: Supplementary file 1 [file Table_1.DOCX]

**Supplementary Online Contents**

eTable 1. Reporting checklist for systematic review (with a meta-analysis).

eTable 2. MOOSE checklist for meta-analyses of observational Studies.

eTable 3. Search terms and number of records.

eTable 4. Main characteristics of the studies conducted on plant-based diets and cancer risk included in the meta-analysis.

eTable 5. Food composition of plant-based diets.

eTable 6. Risk of Bias in Non-randomized Studies of Interventions (ROBINS-I).

# **eTable 1. Reporting checklist for systematic review (with or without a meta-analysis).**

Based on the PRISMA guidelines.

## Instructions to authors

Complete this checklist by entering the page numbers from your manuscript where readers will find each of the items listed below.

Your article may not currently address all the items on the checklist. Please modify your text to include the missing information. If you are certain that an item does not apply, please write "n/a" and provide a short explanation.

Upload your completed checklist as an extra file when you submit to a journal.

In your methods section, say that you used the PRISMAreporting guidelines, and cite them as:

Page MJ, McKenzie JE, Bossuyt PM, Boutron I, Hoffmann TC, Mulrow CD, Shamseer L, Tetzlaff JM, Akl EA, Brennan SE, Chou R, Glanville J, Grimshaw JM, Hróbjartsson A, Lalu MM, Li T, Loder EW, Mayo-Wilson E, McDonald S, McGuinness LA, Stewart LA, Thomas J, Tricco AC, Welch VA, Whiting P, Moher D. The PRISMA 2020 statement: An updated guideline for reporting systematic reviews

|  |  | **Reporting Item** | **Page Number** |
| --- | --- | --- | --- |
| **Title** |  |  |  |
| Title | [#1](https://www.goodreports.org/reporting-checklists/prisma/info/#1) | Identify the report as a systematic review | 1 |
| **Abstract** |  |  |  |
| Abstract | [#2](https://www.goodreports.org/reporting-checklists/prisma/info/#2) | Report an abstract addressing each item in the PRISMA 2020 for Abstracts checklist | 2-3 |
| **Introduction** |  |  |  |
| Background/rationale | [#3](https://www.goodreports.org/reporting-checklists/prisma/info/#3) | Describe the rationale for the review in the context of existing knowledge | 4-6 |
| Objectives | [#4](https://www.goodreports.org/reporting-checklists/prisma/info/#4) | Provide an explicit statement of the objective(s) or question(s) the review addresses | 5-6 |
| **Methods** |  |  |  |
| Eligibility criteria | [#5](https://www.goodreports.org/reporting-checklists/prisma/info/#5) | Specify the inclusion and exclusion criteria for the review and how studies were grouped for the syntheses | 6-7 |
| Information sources | [#6](https://www.goodreports.org/reporting-checklists/prisma/info/#6) | Specify all databases, registers, websites, organisations, reference lists, and other sources searched or consulted to identify studies. Specify the date when each source was last searched or consulted | 6 |
| Search strategy | [#7](https://www.goodreports.org/reporting-checklists/prisma/info/#7) | Present the full search strategies for all databases, registers, and websites, including any filters and limits used | 6, eTable3 |
| Selection process | [#8](https://www.goodreports.org/reporting-checklists/prisma/info/#8) | Specify the methods used to decide whether a study met the inclusion criteria of the review, including how many reviewers screened each record and each report retrieved, whether they worked independently, and, if applicable, details of automation tools used in the process | 7 |
| Data collection process | [#9](https://www.goodreports.org/reporting-checklists/prisma/info/#9) | Specify the methods used to collect data from reports, including how many reviewers collected data from each report, whether they worked independently, any processes for obtaining or confirming data from study investigators, and, if applicable, details of automation tools used in the process | 7 |
| Data items | [#10a](https://www.goodreports.org/reporting-checklists/prisma/info/#10a) | List and define all outcomes for which data were sought. Specify whether all results that were compatible with each outcome domain in each study were sought (for example, for all measures, time points, analyses), and, if not, the methods used to decide which results to collect | 7 |
| Data items | [#10b](https://www.goodreports.org/reporting-checklists/prisma/info/#10b) | List and define all other variables for which data were sought (such as participant and intervention characteristics, funding sources). Describe any assumptions made about any missing or unclear information | eTable4 |
| Study risk of bias assessment | [#11](https://www.goodreports.org/reporting-checklists/prisma/info/#11) | Specify the methods used to assess risk of bias in the included studies, including details of the tool(s) used, how many reviewers assessed each study and whether they worked independently, and, if applicable, details of automation tools used in the process | 8-9 |
| Effect measures | [#12](https://www.goodreports.org/reporting-checklists/prisma/info/#12) | Specify for each outcome the effect measure(s) (such as risk ratio, mean difference) used in the synthesis or presentation of results | 8-9 |
| Synthesis methods | [#13a](https://www.goodreports.org/reporting-checklists/prisma/info/#13a) | Describe the processes used to decide which studies were eligible for each synthesis (such as tabulating the study intervention characteristics and comparing against the planned groups for each synthesis (item #5)) | 8-9 |
| Synthesis methods | [#13b](https://www.goodreports.org/reporting-checklists/prisma/info/#13b) | Describe any methods required to prepare the data for presentation or synthesis, such as handling of missing summary statistics or data conversions | 8-9 |
| Synthesis methods | [#13c](https://www.goodreports.org/reporting-checklists/prisma/info/#13c) | Describe any methods used to tabulate or visually display results of individual studies and syntheses | 8-9 |
| Synthesis methods | [#13d](https://www.goodreports.org/reporting-checklists/prisma/info/#13d) | Describe any methods used to synthesize results and provide a rationale for the choice(s). If meta-analysis was performed, describe the model(s), method(s) to identify the presence and extent of statistical heterogeneity, and software package(s) used | 8-9 |
| Synthesis methods | [#13e](https://www.goodreports.org/reporting-checklists/prisma/info/#13e) | Describe any methods used to explore possible causes of heterogeneity among study results (such as subgroup analysis, meta-regression) | 9 |
| Synthesis methods | [#13f](https://www.goodreports.org/reporting-checklists/prisma/info/#13f) | Describe any sensitivity analyses conducted to assess robustness of the synthesized results | 9 |
| Reporting bias assessment | [#14](https://www.goodreports.org/reporting-checklists/prisma/info/#14) | Describe any methods used to assess risk of bias due to missing results in a synthesis (arising from reporting biases) | 9 |
| Certainty assessment | [#15](https://www.goodreports.org/reporting-checklists/prisma/info/#15) | Describe any methods used to assess certainty (or confidence) in the body of evidence for an outcome | 9 |
| **Results** |  |  |  |
| Study selection | [#16a](https://www.goodreports.org/reporting-checklists/prisma/info/#16a) | Describe the results of the search and selection process, from the number of records identified in the search to the number of studies included in the review, ideally using a flow diagram (http://www.prisma-statement.org/PRISMAStatement/FlowDiagram) | Figure 1 |
| Study selection | [#16b](https://www.goodreports.org/reporting-checklists/prisma/info/#16b) | Cite studies that might appear to meet the inclusion criteria, but which were excluded, and explain why they were excluded | Figure 1 |
| Study characteristics | [#17](https://www.goodreports.org/reporting-checklists/prisma/info/#17) | Cite each included study and present its characteristics | 10-11 |
| Risk of bias in studies | [#18](https://www.goodreports.org/reporting-checklists/prisma/info/#18) | Present assessments of risk of bias for each included study | 11 |
| Results of individual studies | [#19](https://www.goodreports.org/reporting-checklists/prisma/info/#19) | For all outcomes, present for each study (a) summary statistics for each group (where appropriate) and (b) an effect estimate and its precision (such as confidence/credible interval), ideally using structured tables or plots | Table 1 |
| Results of syntheses | [#20a](https://www.goodreports.org/reporting-checklists/prisma/info/#20a) | For each synthesis, briefly summarise the characteristics and risk of bias among contributing studies | 11-12, Table 1 |
| Results of syntheses | [#20b](https://www.goodreports.org/reporting-checklists/prisma/info/#20b) | Present results of all statistical syntheses conducted. If meta-analysis was done, present for each the summary estimate and its precision (such as confidence/credible interval) and measures of statistical heterogeneity. If comparing groups, describe the direction of the effect | 11-12, Table 1 |
| Results of syntheses | [#20c](https://www.goodreports.org/reporting-checklists/prisma/info/#20c) | Present results of all investigations of possible causes of heterogeneity among study results | 11-15 |
| Results of syntheses | [#20d](https://www.goodreports.org/reporting-checklists/prisma/info/#20d) | Present results of all sensitivity analyses conducted to assess the robustness of the synthesised results | 13 |
| Risk of reporting biases in syntheses | [#21](https://www.goodreports.org/reporting-checklists/prisma/info/#21) | Present assessments of risk of bias due to missing results (arising from reporting biases) for each synthesis assessed | 13 |
| Certainty of evidence | [#22](https://www.goodreports.org/reporting-checklists/prisma/info/#22) | Present assessments of certainty (or confidence) in the body of evidence for each outcome assessed | 11-15 |
| **Discussion** |  |  |  |
| Results in context | [#23a](https://www.goodreports.org/reporting-checklists/prisma/info/#23a) | Provide a general interpretation of the results in the context of other evidence | 16-19 |
| Limitations of included studies | [#23b](https://www.goodreports.org/reporting-checklists/prisma/info/#23b) | Discuss any limitations of the evidence included in the review | 18-19 |
| Limitations of the review methods | [#23c](https://www.goodreports.org/reporting-checklists/prisma/info/#23c) | Discuss any limitations of the review processes used | 19 |
| Implications | [#23d](https://www.goodreports.org/reporting-checklists/prisma/info/#23d) | Discuss implications of the results for practice, policy, and future research | 19-20 |
| **Other information** |  |  |  |
| Registration and protocol | [#24a](https://www.goodreports.org/reporting-checklists/prisma/info/#24a) | Provide registration information for the review, including register name and registration number, or state that the review was not registered | 6 |
| Registration and protocol | [#24b](https://www.goodreports.org/reporting-checklists/prisma/info/#24b) | Indicate where the review protocol can be accessed, or state that a protocol was not prepared | 6 |
| Registration and protocol | [#24c](https://www.goodreports.org/reporting-checklists/prisma/info/#24c) | Describe and explain any amendments to information provided at registration or in the protocol | NA |
| Support | [#25](https://www.goodreports.org/reporting-checklists/prisma/info/#25) | Describe sources of financial or non-financial support for the review, and the role of the funders or sponsors in the review | 20 |
| Competing interests | [#26](https://www.goodreports.org/reporting-checklists/prisma/info/#26) | Declare any competing interests of review authors | 20 |
| Availability of data, code, and other materials | [#27](https://www.goodreports.org/reporting-checklists/prisma/info/#27) | Report which of the following are publicly available and where they can be found: template data collection forms; data extracted from included studies; data used for all analyses; analytic code; any other materials used in the review | 21 |

None The PRISMA checklist is distributed under the terms of the Creative Commons Attribution License CC-BY. This checklist can be completed online using <https://www.goodreports.org/>, a tool made by the [EQUATOR Network](https://www.equator-network.org) in collaboration with Penelope.ai.

**eTable 2. MOOSE checklist for meta-analyses of observational studies.**

| **Item No** | **Recommendation** | **Reported on Page No** |
| --- | --- | --- |
| **Reporting of background should include** | | |
| 1 | Problem definition | 3 |
| 2 | Hypothesis statement | 3-5 |
| 3 | Description of study outcome(s) | 3 |
| 4 | Type of exposure or intervention used | 3 |
| 5 | Type of study designs used | 3 |
| 6 | Study population | 3 |
| **Reporting of search strategy should include** | | |
| 7 | Qualifications of searchers (eg. librarians and investigators) | 6 |
| 8 | Search strategy, including time period included in the synthesis and key words | eTabe3 |
| 9 | Effort to include all available studies, including contact with authors | 6 |
| 10 | Databases and registries searched | 6 |
| 11 | Search software used, name and version, including special features used (eg. explosion) | 6 |
| 12 | Use of hand searching (eg. reference lists of obtained articles) | eTable 3 |
| 13 | List of citations located and those excluded, including justification | 7, eTable 3 |
| 14 | Method of addressing articles published in languages other than English | 7 |
| 15 | Method of handling abstracts and unpublished studies | 7 |
| 16 | Description of any contact with authors | 7 |
| **Reporting of methods should include** | | |
| 17 | Description of relevance or appropriateness of studies assembled for assessing the hypothesis to be tested | 7 |
| 18 | Rationale for the selection and coding of data (eg. sound clinical principles or convenience) | 8-9 |
| 19 | Documentation of how data were classified and coded (eg. multiple raters, blinding and interrater reliability) | 8-9 |
| 20 | Assessment of confounding (eg. comparability of cases and controls in studies where appropriate) | 8 |
| 21 | Assessment of study quality, including blinding of quality assessors, stratification or regression on possible predictors of study results | 8 |
| 22 | Assessment of heterogeneity | 9 |
| 23 | Description of statistical methods (eg. complete description of fixed or random effects models, justification of whether the chosen models account for predictors of study results, dose-response models, or cumulative meta-analysis) in sufficient detail to be replicated | 9 |
| 24 | Provision of appropriate tables and graphics | Supplementary Materials |
| **Reporting of results should include** | | |
| 25 | Graphic summarizing individual study estimates and overall estimate | Table 1, 10-15 |
| 26 | Table giving descriptive information for each study included | eTable 4, 10-11 |
| 27 | Results of sensitivity testing (eg. subgroup analysis) | Table 1 |
| 28 | Indication of statistical uncertainty of findings | 13-15 |
| **Reporting of discussion should include** | | |
| 29 | Quantitative assessment of bias (eg. publication bias) | eTable 6 |
| 30 | Justification for exclusion (eg. exclusion of non-English language citations) | 6-7, Figure1 |
| 31 | Assessment of quality of included studies | eTable 6 |
| **Reporting of conclusions should include** | | |
| 32 | Consideration of alternative explanations for observed results | 16-18 |
| 33 | Generalization of the conclusions (ie. appropriate for the data presented and within the domain of the literature review) | 16-18 |
| 34 | Guidelines for future research | 19 |
| 35 | Disclosure of funding source | 20 |

**eTable 3. Search terms and number of records.**

| **PubMed (N = 1286)** |
| --- |
| (cancer OR neoplasm OR tumor OR tumors OR cancers OR neoplasia OR neoplasias OR malignancy OR malignancies OR malignant OR neoplasms OR malignant OR neoplasm OR breast neoplasms OR breast cancer OR breast tumor OR breast tumors OR breast carcinoma OR breast carcinomas OR colorectal neoplasm OR colorectal tumors OR colorectal tumor OR colorectal cancer OR colorectal cancers OR colorectal carcinoma OR colorectal carcinomas OR prostate neoplasms OR prostate neoplasm OR prostatic neoplasm OR prostate cancers OR cancer of the prostate OR prostatic cancer OR prostatic cancers OR cancer of prostate) [Title], AND (vegetarian OR vegetarianism OR vegetarian diet OR dietary pattern OR healthy dietary patterns OR plant-based diets OR healthy diets OR traditional diets OR vegetarian diets OR vegan diet OR vegan OR vegan diets OR plant-based diets OR meatless diets OR lacto-vegetarian OR lactovegetarian OR ovo-vegetarian OR ovovegetarian OR lacto-ovo-vegetarian OR lactoovovegetarian OR pesco-vegetarian OR pescovegetarian OR semi-vegetarian OR semivegetarian) [Title/Abstract] |
| **Web of Science (N = 632)** |
| TI= (cancer OR neoplasm OR tumor OR tumors OR cancers OR neoplasia OR neoplasias OR malignancy OR malignancies OR malignant OR neoplasms OR malignant OR neoplasm OR “breast neoplasms” OR “breast cancer” OR “breast tumor” OR “breast tumors” OR “breast carcinoma” OR “breast carcinomas” OR “colorectal neoplasm” OR “colorectal tumors” OR “colorectal tumor” OR “colorectal cancer” OR “colorectal cancers” OR “colorectal carcinoma” OR “colorectal carcinomas” OR “prostate neoplasms” OR “prostate neoplasm” OR “prostatic neoplasm” OR “prostate cancers” OR “cancer of the prostate” OR “prostatic cancer” OR “prostatic cancers” OR “cancer of prostate”) AND TS= (vegetarian OR vegetarianism OR “vegetarian diet” OR “dietary pattern” OR “healthy dietary patterns” OR “plant-based diets” OR “healthy diets” OR “traditional diets” OR “vegetarian diets” OR “vegan diet” OR vegan OR “vegan diets” OR “plant-based diets” OR “meatless diets” OR “lacto-vegetarian” OR lactovegetarian OR “ovo-vegetarian” OR ovovegetarian OR “lacto-ovo-vegetarian” OR lactoovovegetarian OR “pesco-vegetarian” OR pescovegetarian OR “semi-vegetarian” OR semivegetarian) |
| **EMBASE (N = 869)** |
| (cancer:ti OR tumor:ti OR tumors:ti OR cancers:ti OR neoplasia:ti OR neoplasias:ti OR malignancy:ti OR malignancies:ti OR neoplasms:ti OR malignant:ti OR neoplasm:ti OR 'breast neoplasms':ti OR 'breast cancer':ti OR 'breast tumor':ti OR 'breast tumors':ti OR 'breast carcinoma':ti OR 'breast carcinomas':ti OR 'colorectal neoplasm':ti OR 'colorectal tumors':ti OR 'colorectal tumor':ti OR 'colorectal cancer':ti OR 'colorectal cancers':ti OR 'colorectal carcinoma':ti OR 'colorectal carcinomas':ti OR 'prostate neoplasms':ti OR 'prostate neoplasm':ti OR 'prostatic neoplasm':ti OR 'prostate cancers':ti OR 'cancer of the prostate':ti OR 'prostatic cancer':ti OR 'prostatic cancers':ti OR 'cancer of prostate':ti AND (vegetarian:ab,ti OR vegetarianism:ti OR 'vegetarian diet':ab,ti OR 'dietary pattern':ab,ti OR 'healthy dietary patterns':ab,ti OR 'healthy diets':ab,ti OR 'vegetarian diets':ab,ti OR 'vegan diet':ab,ti OR vegan:ab,ti OR 'vegan diets':ab,ti OR 'plant-based diets':ab,ti OR 'meatless diets':ab,ti OR 'lacto-vegetarian':ab,ti OR lactovegetarian:ab,ti OR 'ovo-vegetarian':ab,ti OR ovovegetarian:ab,ti OR 'lacto-ovo-vegetarian':ab,ti OR lactoovovegetarian:ab,ti OR 'pesco-vegetarian':ab,ti OR pescovegetarian:ab,ti OR 'semi-vegetarian':ab,ti OR semivegetarian:ab,ti |
| **Scopus (N = 2445)** |
| ((TITLE (cancer) OR TITLE (neoplasm) OR TITLE (tumor) OR TITLE (tumors) OR TITLE (cancers) OR TITLE (neoplasia) OR TITLE (neoplasias) OR TITLE (malignancy) OR TITLE (malignancies) OR TITLE (malignant) OR TITLE (neoplasms) OR TITLE (malignant) OR TITLE (neoplasm) OR TITLE ("breast neoplasms") OR TITLE ("breast cancer") OR TITLE ("breast tumor") OR TITLE ("breast tumors") OR TITLE ("breast carcinoma") OR TITLE ("breast carcinomas") OR TITLE ("colorectal neoplasm") OR TITLE ("colorectal tumors") OR TITLE ("colorectal tumor") OR TITLE ("colorectal cancer") OR TITLE ("colorectal cancers") OR TITLE ("colorectal carcinoma") OR TITLE ("colorectal carcinomas") OR TITLE ("prostate neoplasms") OR TITLE ("prostate neoplasm") OR TITLE ("prostatic neoplasm") OR TITLE ("prostate cancers") OR TITLE ("cancer of the prostate") OR TITLE ("prostatic cancer") OR TITLE ("prostatic cancers") OR TITLE ("cancer of prostate"))) AND ((TITLE (vegetarian) OR TITLE (vegetarianism) OR TITLE ("vegetarian diet") OR TITLE ("dietary pattern") OR TITLE ("healthy dietary patterns") OR TITLE ("plant-based diets") OR TITLE ("healthy diets") OR TITLE ("traditional diets") OR TITLE ("vegetarian diets") OR TITLE ("vegan diet") OR TITLE (vegan) OR TITLE ("vegan diets") OR TITLE ("plant-based diets") OR TITLE ("meatless diets") OR TITLE ("lacto-vegetarian") OR TITLE (lactovegetarian) OR TITLE ("ovo-vegetarian") OR TITLE (ovovegetarian) OR TITLE ("lacto-ovo-vegetarian") OR TITLE (lactoovovegetarian) OR TITLE ("colorectal tumors") OR TITLE ("pesco-vegetarian") OR TITLE (pescovegetarian) OR TITLE ("semi-vegetarian") OR TITLE (semivegetarian))) |

**eTable 4.1 Main characteristics of the studies conducted on plant-based diets and cancer risk included in the meta-analysis.**

| **Year** | **Author** | **Study type** | **Country** | **Follow up** | **Age** | **Cancer site** | **Total population** | **Case number** | **Sex** | **Vegetarian type** | **Frequency/ Score** | **RR (95%CI)** |
| --- | --- | --- | --- | --- | --- | --- | --- | --- | --- | --- | --- | --- |
| 1988 | Mills | cohort study | USA | 6 | >25 | pancreas | 95196 | 40 | Both male and female | Vegan | l-2/week | 0.29 (0.10-0.84) |
| 1988 | Mills | cohort study | USA | 6 | >25 | pancreas | 95196 | 40 | Both male and female | Vegan | ＞=3/week | 0.40 (0.18-0.91) |
| 1988 | Mills | cohort study | USA | 6 | >25 | pancreas | 95196 | 40 | Both male and female | Vegan | l-2/week | 0.47 (0.21-1.02) |
| 1988 | Mills | cohort study | USA | 6 | >25 | pancreas | 95196 | 40 | Both male and female | Vegan | ＞=3/week | 0.43 (0.16-1.13) |
| 1988 | Mills | cohort study | USA | 6 | >25 | pancreas | 95196 | 40 | Both male and female | Vegan | l-2/week | 0.47 (0.19-1.15) |
| 1988 | Mills | cohort study | USA | 6 | >25 | pancreas | 95196 | 40 | Both male and female | Vegan | ＞=3/week | 0.35 (0.17-0.73) |
| 1994 | Centonze | case-control study | Italy | NA | 69 | colorectum | 95196 | 40 | Both male and female | Vegan | NA | 0.51 (0.25-1.04) |
| 1998 | Slattery | case-control study | USA | NA | 30-79 | colon | 95196 | 40 | NA | Prudent diet | NA | 0.66 (0.50-0.86) |
| 1998 | Slattery | case-control study | USA | NA | <67 | colon | 95196 | 40 | NA | Prudent diet | NA | 0.63 (0.43-0.92) |
| 1998 | Slattery | case-control study | USA | NA | ≥67 | colon | 218865 | 132 | NA | Prudent diet | NA | 0.67 (0.45-1.00) |
| 2001 | Terry | cohort study | Sweden | 9.6 | 50 | colorectum | 61463 | 460 | Female | Semi vegetarian | NA | 0.79 (0.56-1.10) |
| 2001 | Terry | cohort study | Sweden | 9.6 | 50 | colon | 61463 | 460 | Female | Semi vegetarian | NA | 0.83 (0.54-1.26) |
| 2001 | Terry | cohort study | Sweden | 9.6 | 50 | rectum | 61463 | 460 | Female | Semi vegetarian | NA | 0.77 (0.44-1.35) |
| 2003 | Masaki | cohort study | Japan | 10 | 40-79 | stomach | 59000 | 620 | Male | Vegan | NA | 1.06 (0.61-1.87) |
| 2003 | Masaki | cohort study | Japan | 10 | 40-79 | stomach | 59000 | 620 | Male | Vegan | NA | 0.78 (0.42-1.44) |
| 2004 | Wu | cohort study | USA | 15 | 55.7 | colon | 5765 | 310 | Male | Prudent diet | 2 | 0.82 (0.63-1.08) |
| 2004 | Wu | cohort study | USA | 15 | 55.7 | colon | 5765 | 310 | Male | Prudent diet | 3 | 0.80 (0.61-1.03) |
| 2004 | Wu | cohort study | USA | 15 | 55.7 | colon | 5765 | 310 | Male | Prudent diet | 4 | 0.86 (0.66-1.12) |
| 2004 | Wu | cohort study | USA | 15 | 55.7 | colon | 51129 | 561 | Male | Prudent diet | 5 | 0.84 (0.64-1.10) |
| 2005 | Kim | cohort study | Japan | 10 | 40-59 | colorectum | 51129 | 561 | Male | Pesco-vegetarian | NA | 0.88 (0.60-1.30) |
| 2005 | Kim | cohort study | Japan | 10 | 40-59 | colorectum | 51129 | 561 | Male | Pesco-vegetarian | NA | 1.01 (0.69-1.48) |
| 2005 | Kim | cohort study | Japan | 10 | 40-59 | colorectum | 51129 | 561 | Male | Pesco-vegetarian | NA | 0.81 (0.52-1.24) |
| 2005 | Kim | cohort study | Japan | 10 | 40-59 | colorectum | 51129 | 561 | Female | Pesco-vegetarian | NA | 0.94 (0.58-1.51) |
| 2005 | Kim | cohort study | Japan | 10 | 40-59 | colorectum | 42112 | 370 | Female | Pesco-vegetarian | NA | 0.80 (0.48-1.32) |
| 2005 | Kim | cohort study | Japan | 10 | 40-59 | colorectum | 42112 | 370 | Female | Pesco-vegetarian | NA | 0.98 (0.58-1.65) |
| 2005 | Kim | cohort study | Japan | 10 | 40-59 | colon | 42112 | 370 | Male | Pesco-vegetarian | NA | 0.97 (0.60-1.54) |
| 2005 | Kim | cohort study | Japan | 10 | 40-59 | colon | 42112 | 370 | Male | Pesco-vegetarian | NA | 1.13 (0.71-1.80) |
| 2005 | Kim | cohort study | Japan | 10 | 40-59 | colon | 42112 | 370 | Male | Pesco-vegetarian | NA | 0.83 (0.49-1.41) |
| 2005 | Kim | cohort study | Japan | 10 | 40-59 | colon | 42112 | 370 | Female | Pesco-vegetarian | NA | 0.88 (0.48-1.60) |
| 2005 | Kim | cohort study | Japan | 10 | 40-59 | colon | 42112 | 370 | Female | Pesco-vegetarian | NA | 1.02 (0.57-1.85) |
| 2005 | Kim | cohort study | Japan | 10 | 40-59 | colon | 42112 | 370 | Female | Pesco-vegetarian | NA | 0.76 (0.39-1.50) |
| 2005 | Kim | cohort study | Japan | 10 | 40-59 | rectum | 42112 | 370 | Male | Pesco-vegetarian | NA | 0.74 (0.38-1.43) |
| 2005 | Kim | cohort study | Japan | 10 | 40-59 | rectum | 42112 | 370 | Male | Pesco-vegetarian | NA | 0.79 (0.40-1.54) |
| 2005 | Kim | cohort study | Japan | 10 | 40-59 | rectum | 42112 | 370 | Male | Pesco-vegetarian | NA | 0.76 (0.37-1.58) |
| 2005 | Kim | cohort study | Japan | 10 | 40-59 | rectum | 42112 | 370 | Female | Pesco-vegetarian | NA | 1.05 (0.48-2.30) |
| 2005 | Kim | cohort study | Japan | 10 | 40-59 | rectum | 42112 | 370 | Female | Pesco-vegetarian | NA | 0.33 (0.11-1.05) |
| 2005 | Kim | cohort study | Japan | 10 | 40-59 | rectum | 42112 | 370 | Female | Pesco-vegetarian | NA | 1.43 (0.62-3.28) |
| 2005 | Nkondjock | case-control study | Canada | NA | 30-74 | pancreas | 42112 | 370 | Male | Vegan | NA | 0.51 (0.29-0.90) |
| 2005 | Nkondjock | case-control study | Canada | NA | 30-74 | pancreas | 42112 | 370 | Female | Vegan | NA | 1.06 (0.57-1.96) |
| 2005 | Rouillier | case-control study | France | NA | 30-79 | colorectum | 42112 | 370 | Both male and female | Vegan | NA | 1.40 (0.60-3.10) |
| 2008 | Campbell | case-control study | Canada | NA | NA | stomach | 42112 | 370 | Female | Prudent diet | NA | 0.58 (0.37-0.92) |
| 2008 | Campbell | case-control study | Canada | NA | 20-74 | stomach | 42112 | 370 | Male | Prudent diet | NA | 1.06 (0.77-1.45) |
| 2009 | Wirfalt | cohort study | USA | 4.5 | 50-71 | rectum | 42112 | 370 | Male | Semi vegetarian | NA | 0.89 (0.79-1.01) |
| 2009 | Wirfalt | cohort study | USA | 4.5 | 50-71 | rectum | 42112 | 370 | Male | Semi vegetarian | NA | 0.74 (0.60-1.17) |
| 2009 | Wirfalt | cohort study | USA | 4.5 | 50-71 | rectum | 42112 | 370 | Female | Semi vegetarian | NA | 0.90 (0.75-1.08) |
| 2009 | Wirfalt | cohort study | USA | 4.5 | 50-71 | rectum | 42112 | 370 | Female | Semi vegetarian | NA | 0.95 (0.69-1.29) |
| 2009 | Joshi | case-control study | India | NA | 54.6 | oesophagus | 42112 | 370 | Both male and female | Vegan | NA | 0.66 (0.47-1.49) |
| 2009 | Joshi | case-control study | India | NA | 54.6 | oesophagus | 5669 | 630 | Both male and female | Vegan | NA | 0.26 (0.15-2.15) |
| 2009 | Joshi | case-control study | India | NA | 54.6 | oesophagus | 5669 | 630 | Both male and female | Vegan | NA | 0.55 (0.42-1.42) |
| 2009 | Joshi | case-control study | India | NA | 54.6 | oesophagus | 1372 | 171 | Both male and female | Vegan | NA | 0.36 (0.26-1.62) |
| 2009 | Joshi | case-control study | India | NA | 54.6 | oesophagus | 3501 | 1169 | Both male and female | Vegan | NA | 0.80 (0.22-3.84) |
| 2009 | Joshi | case-control study | India | NA | 54.6 | oesophagus | 3501 | 1169 | Both male and female | Vegan | NA | 0.79 (0.23-3.51) |
| 2010 | Kurotani | case-control study | Japan | NA | 20-74 | colorectum | 492306 | 2170 | Both male and female | Prudent diet | NA | 0.79 (0.58-1.08) |
| 2010 | Buckland | cohort study | Europe | 8.9 | 35-70 | stomach | 492306 | 2170 | NA | Mediterranean diet | 7-10 | 0.82 (0.64-1.04) |
| 2010 | Buckland | cohort study | Europe | 8.9 | 35-70 | stomach | 492306 | 2170 | NA | Mediterranean diet | 11-18 | 0.67 (0.47-0.94) |
| 2010 | Miller | case-control study | USA | NA | 66 | colorectum | 492306 | 2170 | Male | Vegan | NA | 0.38 (0.21-0.69) |
| 2010 | Miller | case-control study | USA | NA | 66 | colorectum | 492306 | 965 | Male | Vegan | NA | 0.35 (0.19-0.65) |
| 2011 | Magalha˜es | case-control study | Portugal | NA | 64 | colorectum | 492306 | 965 | Both male and female | Semi vegetarian | NA | 1.76 (1.09-2.85) |
| 2011 | Magalha˜es | case-control study | Portugal | NA | 64 | colon | 492306 | 965 | Both male and female | Semi vegetarian | NA | 2.35 (1.19-4.64) |
| 2011 | Magalha˜es | case-control study | Portugal | NA | 64 | rectum | 492306 | 965 | Both male and female | Semi vegetarian | NA | 1.41 (0.75-2.63) |
| 2011 | Reedy | cohort study | USA | 5 | 50-71 | colorectum | 780 | 94 | Male | Vegan | NA | 0.85 (0.76-0.94) |
| 2011 | Reedy | cohort study | USA | 5 | 50-71 | colorectum | 780 | 94 | Female | Vegan | NA | 0.90 (0.77-1.06) |
| 2012 | Bradshaw | case-control study | USA | NA | 20-80 | oral/pharynx | 780 | 94 | Both male and female | Semi vegetarian | NA | 0.53 (0.39-0.71) |
| 2012 | Bradshaw | case-control study | USA | NA | 20-80 | oral/pharynx | 780 | 94 | Both male and female | Semi vegetarian | NA | 0.45 (0.32-0.63) |
| 2012 | Bradshaw | case-control study | USA | NA | 20-80 | larynx | 780 | 94 | Both male and female | Semi vegetarian | NA | 0.73 (0.48-1.10) |
| 2012 | Ibiebele | case-control study | Australia | NA | 18-79 | oesophagus | 780 | 94 | Both male and female | Vegan | NA | 1.29 (0.77-2.14) |
| 2012 | Ibiebele | case-control study | Australia | NA | 18-79 | oesophagus | 780 | 94 | Both male and female | Vegan | NA | 0.66 (0.42-1.04) |
| 2012 | Ibiebele | case-control study | Australia | NA | 18-79 | oesophagus | 780 | 94 | Both male and female | Vegan | NA | 0.41 (0.24-0.70) |
| 2013 | Praud | case-control study | Italy | NA | 61 | stomach | 780 | 94 | Both male and female | Mediterranean diet | 4 | 0.78 (0.63-0.96) |
| 2013 | Praud | case-control study | Italy | NA | 61 | stomach | 1575 | 800 | Both male and female | Mediterranean diet | 5 | 0.61 (0.49-0.77) |
| 2013 | Praud | case-control study | Italy | NA | 61 | stomach | 485044 | 449 | Both male and female | Mediterranean diet | ≥6 | 0.57 (0.45-0.70) |
| 2013 | Safar | case-control study | Iran | NA | 40-75 | colorectum | 485044 | 449 | Both male and female | Pesco-laco-ovo-vegetarian | NA | 0.23 (0.11-0.48) |
| 2013 | Kontou | case-control study | Greece | NA | 63 | colorectum | 485044 | 449 | Both male and female | Mediterranean diet | NA | 0.87 (0.82-0.92) |
| 2013 | Chan | case-control study | USA | NA | 21-85 | pancreas | 1157 | 431 | Male | Prudent diet | NA | 0.51 (0.31-0.84) |
| 2013 | Chan | case-control study | USA | NA | 21-85 | pancreas | 1157 | 431 | Female | Prudent diet | NA | 0.51 (0.29-0.90) |
| 2013 | Bosetti | case-control study | Italy | NA | 62 | pancreas | 1132 | 253 | Both male and female | Mediterranean diet | 3 | 0.93 (0.71-1.23) |
| 2013 | Bosetti | case-control study | Italy | NA | 62 | pancreas | 1132 | 253 | Both male and female | Mediterranean diet | 4 | 0.66 (0.50-0.88) |
| 2013 | Bosetti | case-control study | Italy | NA | 62 | pancreas | 1132 | 253 | Both male and female | Mediterranean diet | 5 | 0.57 (0.42-0.77) |
| 2013 | Bosetti | case-control study | Italy | NA | 62 | pancreas | 492306 | 3110 | Both male and female | Mediterranean diet | ≥6 | 0.48 (0.35-0.67) |
| 2013 | Arem | cohort study | USA | 10.5 | 63 | pancreas | 492306 | 3110 | Both male and female | Semi vegetarian | NA | 0.85 (0.74-0.97) |
| 2013 | Arem | cohort study | USA | 10.5 | 63 | pancreas | 2493 | 1176 | Male | Semi vegetarian | NA | 0.83 (0.74-0.97) |
| 2013 | Arem | cohort study | USA | 10.5 | 63 | pancreas | 2493 | 1176 | Female | Semi vegetarian | NA | 0.87 (0.70-1.09) |
| 2013 | Agnoli | cohort study | Italy | 11.28 | NA | colorectum | 2493 | 1176 | Both male and female | Mediterranean diet | 2-3 | 0.49 (0.35-0.67) |
| 2013 | Agnoli | cohort study | Italy | 11.28 | NA | colorectum | 2387 | 880 | Both male and female | Mediterranean diet | 4-5 | 0.58 (0.42-0.79) |
| 2013 | Agnoli | cohort study | Italy | 11.28 | NA | colorectum | 2387 | 880 | Both male and female | Mediterranean diet | 6-11 | 0.50 (0.35-0.71) |
| 2013 | Agnoli | cohort study | Italy | 11.28 | NA | colorectum | 2387 | 880 | Male | Mediterranean diet | 2-3 | 0.70 (0.42-1.17) |
| 2013 | Agnoli | cohort study | Italy | 11.28 | NA | colorectum | 3627 | 999 | Male | Mediterranean diet | 4-5 | 0.72 (0.44-1.19) |
| 2013 | Agnoli | cohort study | Italy | 11.28 | NA | colorectum | 3627 | 999 | Male | Mediterranean diet | 6-11 | 0.54 (0.30-0.96) |
| 2013 | Agnoli | cohort study | Italy | 11.28 | NA | colorectum | 3627 | 999 | Female | Mediterranean diet | 2-3 | 0.36 (0.23-0.55) |
| 2013 | Agnoli | cohort study | Italy | 11.28 | NA | colorectum | 3627 | 999 | Female | Mediterranean diet | 4-5 | 0.49 (0.33-0.73) |
| 2013 | Agnoli | cohort study | Italy | 11.28 | NA | colorectum | 267 | 89 | Female | Mediterranean diet | 6-11 | 0.46 (0.30-0.72) |
| 2013 | Bosetti2 | case-control study | Italy | NA | NA | pancreas | 500 | 250 | Both male and female | Vegan | 2 | 0.93 (0.63-1.39) |
| 2013 | Bosetti2 | case-control study | Italy | NA | NA | pancreas | 2233 | 532 | Both male and female | Vegan | 3 | 0.68 (0.44-1.04) |
| 2013 | Bosetti2 | case-control study | Italy | NA | NA | pancreas | 2233 | 532 | Both male and female | Vegan | 4 | 0.55 (0.35-0.86) |
| 2013 | Zhang | cohort study | China | NA | 40-74 | liver | 2892 | 688 | Both male and female | Vegan | NA | 0.58 (0.40-0.84) |
| 2013 | Zhang | cohort study | China | NA | 40-74 | liver | 2892 | 688 | Male | Vegan | NA | 0.52 (0.31-0.89) |
| 2013 | Zhang | cohort study | China | NA | 40-74 | liver | 2892 | 688 | Female | Vegan | NA | 0.68 (0.40-1.14) |
| 2014 | Kumagai | cohort study | Japan | 11 | 40-79 | colorectum | 2892 | 688 | Both male and female | Vegan | 2 | 0.88 (0.72-1.06) |
| 2014 | Kumagai | cohort study | Japan | 11 | 40-79 | colorectum | 2892 | 688 | Both male and female | Vegan | 3 | 0.82 (0.66-1.03) |
| 2014 | Kumagai | cohort study | Japan | 11 | 40-79 | colorectum | 537218 | 2383 | Both male and female | Vegan | 4 | 0.76 (0.60-0.97) |
| 2014 | Kumagai | cohort study | Japan | 11 | 40-79 | colon | 537218 | 2383 | Both male and female | Vegan | 2 | 0.93 (0.73-1.19) |
| 2014 | Kumagai | cohort study | Japan | 11 | 40-79 | colon | 537218 | 2383 | Both male and female | Vegan | 3 | 0.89 (0.67-1.17) |
| 2014 | Kumagai | cohort study | Japan | 11 | 40-79 | colon | 45275 | 435 | Both male and female | Vegan | 4 | 0.89 (0.66-1.19) |
| 2014 | Kumagai | cohort study | Japan | 11 | 40-79 | rectum | 45275 | 435 | Both male and female | Vegan | 2 | 0.79 (0.58-1.07) |
| 2014 | Kumagai | cohort study | Japan | 11 | 40-79 | rectum | 45275 | 435 | Both male and female | Vegan | 3 | 0.76 (0.53-1.08) |
| 2014 | Kumagai | cohort study | Japan | 11 | 40-79 | rectum | 45275 | 435 | Both male and female | Vegan | 4 | 0.56 (0.37-0.84) |
| 2014 | Grosso | case-control study | Italy | NA | 65.3 | colorectum | 45275 | 435 | Both male and female | Mediterranean diet | medium | 0.53 (0.39-0.74) |
| 2014 | Grosso | case-control study | Italy | NA | 65.3 | colorectum | 45275 | 435 | Both male and female | Mediterranean diet | high | 0.46 (0.28-0.75) |
| 2014 | Denova-Gutiérrez | case-control study | Mexico | NA | 20 | stomach | 45275 | 435 | Both male and female | Semi vegetarian | NA | 0.43 (0.24-0.77) |
| 2014 | Filomeno | case-control study | Italy | NA | 58 | oral/pharynx | 45275 | 435 | Both male and female | Mediterranean diet | 3 | 0.46 (0.33-0.65) |
| 2014 | Filomeno | case-control study | Italy | NA | 58 | oral/pharynx | 45275 | 435 | Both male and female | Mediterranean diet | 4 | 0.45 (0.32-0.63) |
| 2014 | Filomeno | case-control study | Italy | NA | 58 | oral/pharynx | 45275 | 435 | Both male and female | Mediterranean diet | 6-9 | 0.20 (0.14-0.28) |
| 2015 | Orlich | cohort study | USA | 7.3 | ≥25 | colon | 45275 | 435 | Both male and female | Vegan | NA | 0.83 (0.66-1.05) |
| 2015 | Orlich | cohort study | USA | 7.3 | ≥25 | rectum | 45275 | 435 | Both male and female | Vegan | NA | 0.66 (0.43-1.02) |
| 2015 | Gilsing | cohort study | Netherlands | 20.3 | 55-69 | colorectum | 978 | 326 | Both male and female | Lacto-ovo-vegetarian | NA | 0.89 (0.58-1.39) |
| 2015 | Gilsing | cohort study | Netherlands | 20.3 | 55-69 | colon | 978 | 326 | Both male and female | Lacto-ovo-vegetarian | NA | 0.95 (0.60-1.52) |
| 2015 | Gilsing | cohort study | Netherlands | 20.3 | 55-69 | rectum | 978 | 326 | Both male and female | Lacto-ovo-vegetarian | NA | 0.22 (0.03-1.63) |
| 2015 | McLean | case-control study | Canada | NA | 20-70 | colon | 132837 | 267 | Male | DASH diet | NA | 0.45 (0.25-0.83) |
| 2015 | McLean | case-control study | Canada | NA | 20-70 | colon | 132837 | 267 | Male | DASH diet | 3 | 0.98 (0.68-1.41) |
| 2015 | McLean | case-control study | Canada | NA | 20-70 | colon | 132837 | 267 | Male | DASH diet | 4 | 1.07 (0.76-1.51) |
| 2015 | McLean | case-control study | Canada | NA | 20-70 | colon | 44097 | 854 | Male | DASH diet | 5 | 1.06 (0.75-1.50) |
| 2015 | McLean | case-control study | Canada | NA | 20-70 | colon | 44097 | 854 | Male | DASH diet | 6 | 1.20 (0.85-1.70) |
| 2015 | McLean | case-control study | Canada | NA | 20-70 | colon | 44097 | 854 | Male | DASH diet | 7 | 0.92 (0.63-1.33) |
| 2015 | McLean | case-control study | Canada | NA | 20-70 | colon | 44097 | 854 | Male | DASH diet | 8 | 0.65 (0.44-0.97) |
| 2015 | McLean | case-control study | Canada | NA | 20-70 | colon | 44097 | 854 | Female | DASH diet | 3 | 1.04 (0.69-1.57) |
| 2015 | McLean | case-control study | Canada | NA | 20-70 | colon | 44097 | 854 | Female | DASH diet | 4 | 1.12 (0.76-1.64) |
| 2015 | McLean | case-control study | Canada | NA | 20-70 | colon | 44097 | 854 | Female | DASH diet | 5 | 1.06 (0.72-1.55) |
| 2015 | McLean | case-control study | Canada | NA | 20-70 | colon | 44097 | 854 | Female | DASH diet | 6 | 1.01 (0.67-1.51) |
| 2015 | McLean | case-control study | Canada | NA | 20-70 | colon | 44097 | 854 | Female | DASH diet | 7 | 1.00 (0.66-1.51) |
| 2015 | McLean | case-control study | Canada | NA | 20-70 | colon | 44097 | 854 | Female | DASH diet | 8 | 1.15 (0.76-1.74) |
| 2015 | McLean | case-control study | Canada | NA | 20-70 | rectum | 44097 | 854 | Male | DASH diet | 3 | 1.32 (0.91-1.93) |
| 2015 | McLean | case-control study | Canada | NA | 20-70 | rectum | 44097 | 854 | Male | DASH diet | 4 | 1.57 (1.10-2.25) |
| 2015 | McLean | case-control study | Canada | NA | 20-70 | rectum | 1014 | 338 | Male | DASH diet | 5 | 1.27 (0.88-1.83) |
| 2015 | McLean | case-control study | Canada | NA | 20-70 | rectum | 1014 | 338 | Male | DASH diet | 6 | 1.26 (0.87-1.83) |
| 2015 | McLean | case-control study | Canada | NA | 20-70 | rectum | 1014 | 338 | Male | DASH diet | 7 | 1.01 (0.68-1.50) |
| 2015 | McLean | case-control study | Canada | NA | 20-70 | rectum | 770 | 263 | Male | DASH diet | 8 | 0.64 (0.42-0.98) |
| 2015 | McLean | case-control study | Canada | NA | 20-70 | rectum | 2846 | 768 | Female | DASH diet | 3 | 1.02 (0.66-1.57) |
| 2015 | McLean | case-control study | Canada | NA | 20-70 | rectum | 2846 | 768 | Female | DASH diet | 4 | 0.79 (0.52-1.19) |
| 2015 | McLean | case-control study | Canada | NA | 20-70 | rectum | 2846 | 768 | Female | DASH diet | 5 | 0.98 (0.65-1.47) |
| 2015 | McLean | case-control study | Canada | NA | 20-70 | rectum | 2846 | 768 | Female | DASH diet | 6 | 1.23 (0.81-1.97) |
| 2015 | McLean | case-control study | Canada | NA | 20-70 | rectum | 77659 | 490 | Female | DASH diet | 7 | 0.92 (0.59-1.42) |
| 2015 | McLean | case-control study | Canada | NA | 20-70 | rectum | 77659 | 490 | Female | DASH diet | 8 | 1.03 (0.66-1.60) |
| 2015 | McLean | case-control study | Canada | NA | 20-70 | colorectum | 77659 | 490 | Male | DASH diet | 3 | 1.13 (0.84-1.53) |
| 2015 | McLean | case-control study | Canada | NA | 20-70 | colorectum | 77659 | 490 | Male | DASH diet | 4 | 1.31 (0.98-1.75) |
| 2015 | McLean | case-control study | Canada | NA | 20-70 | colorectum | 10210 | 437 | Male | DASH diet | 5 | 1.17 (0.88-1.57) |
| 2015 | McLean | case-control study | Canada | NA | 20-70 | colorectum | 10210 | 437 | Male | DASH diet | 6 | 1.25 (0.93-1.68) |
| 2015 | McLean | case-control study | Canada | NA | 20-70 | colorectum | 10210 | 307 | Male | DASH diet | 7 | 0.97 (0.71-1.32) |
| 2015 | McLean | case-control study | Canada | NA | 20-70 | colorectum | 10210 | 307 | Male | DASH diet | 8 | 0.66 (0.47-0.92) |
| 2015 | McLean | case-control study | Canada | NA | 20-70 | colorectum | 10210 | 92 | Female | DASH diet | 3 | 1.05 (0.74-1.48) |
| 2015 | McLean | case-control study | Canada | NA | 20-70 | colorectum | 10210 | 92 | Female | DASH diet | 4 | 0.96 (0.70-1.33) |
| 2015 | McLean | case-control study | Canada | NA | 20-70 | colorectum | 6258 | 3161 | Female | DASH diet | 5 | 1.04 (0.75-1.42) |
| 2015 | McLean | case-control study | Canada | NA | 20-70 | colorectum | 6258 | 3161 | Female | DASH diet | 6 | 1.10 (0.79-1.53) |
| 2015 | McLean | case-control study | Canada | NA | 20-70 | colorectum | 6258 | 3161 | Female | DASH diet | 7 | 0.96 (0.68-1.35) |
| 2015 | McLean | case-control study | Canada | NA | 20-70 | colorectum | 6258 | 3161 | Female | DASH diet | 8 | 1.09 (0.77-1.54) |
| 2015 | Azizi | case-control study | Iran | NA | 35-75 | colorectum | 6258 | 3161 | Both male and female | Pesco-laco-ovo-vegetarian | NA | 0.18 (0.09-0.47) |
| 2015 | Chen | case-control study | Canada | NA | 20-74 | colorectum | 6258 | 3161 | Both male and female | Vegan | NA | 0.55 (0.35-0.87) |
| 2016 | Park | case-control study | Korea | NA | 56 | colorectum | 6258 | 3161 | Both male and female | Prudent diet | NA | 0.37 (0.28-0.48) |
| 2016 | Park | case-control study | Korea | NA | 56 | rectum | 6258 | 3161 | Both male and female | Prudent diet | NA | 0.38 (0.27-0.54) |
| 2016 | Park | case-control study | Korea | NA | 56 | colorectum | 6258 | 3161 | Male | Prudent diet | NA | 0.27 (0.19-0.39) |
| 2016 | Park | case-control study | Korea | NA | 56 | rectum | 6258 | 3161 | Male | Prudent diet | NA | 0.21 (0.12-0.36) |
| 2016 | Park | case-control study | Korea | NA | 56 | colorectum | 6258 | 3161 | Female | Prudent diet | NA | 0.33 (0.20-0.54) |
| 2017 | Jones | cohort study | British | 17.4 | 52 | colorectum | 6258 | 3161 | Female | Mediterranean diet | NA | 0.82 (0.57-1.17) |
| 2017 | Jones | cohort study | British | 17.4 | 52 | colon | 6258 | 3161 | Female | Mediterranean diet | NA | 1.03 (0.67-1.57) |
| 2017 | Jones | cohort study | British | 17.4 | 52 | rectum | 6258 | 3161 | Female | Mediterranean diet | NA | 0.38 (0.20-0.74) |
| 2018 | Rada-Fernandez de | cohort study | British | 17.2 | 35-69 | colorectum | 6258 | 3161 | Both male and female | Semi vegetarian | NA | 0.80 (0.58-1.11) |
| 2018 | Rada-Fernandez de | cohort study | British | 17.2 | 35-69 | colon | 6258 | 3161 | Both male and female | Semi vegetarian | NA | 0.71 (0.47-1.08) |
| 2018 | Rada-Fernandez de | cohort study | British | 17.2 | 35-69 | rectum | 6258 | 3161 | Both male and female | Semi vegetarian | NA | 1.03 (0.63-1.66) |
| 2018 | Shin | cohort study | Japan | 13.8 | 40-69 | colorectum | 6258 | 3161 | Both male and female | Prudent diet | NA | 0.85 (0.72-1.00) |
| 2018 | Shin | cohort study | Japan | 13.8 | 40-69 | colon | 6258 | 3161 | Both male and female | Prudent diet | NA | 0.85 (0.70-1.04) |
| 2018 | Shin | cohort study | Japan | 13.8 | 40-69 | rectum | 6258 | 3161 | Both male and female | Prudent diet | NA | 0.84 (0.63-1.13) |
| 2018 | Castelló | case-control study | Spain | NA | 20-85 | stomach | 6258 | 3161 | Both male and female | Prudent diet | NA | 1.40 (0.93-2.11) |
| 2018 | Castelló | case-control study | Spain | NA | 20-85 | stomach | 6258 | 3161 | Both male and female | Mediterranean diet | NA | 0.53 (0.34-0.82) |
| 2018 | Castelló | case-control study | Spain | NA | 20-85 | stomach | 6258 | 3161 | Male | Prudent diet | NA | 1.28 (0.81-2.05) |
| 2018 | Castelló | case-control study | Spain | NA | 20-85 | stomach | 6258 | 3161 | Male | Mediterranean diet | NA | 0.51 (0.31-0.83) |
| 2018 | Castelló | case-control study | Spain | NA | 20-85 | stomach | 6258 | 3161 | Female | Prudent diet | NA | 1.96 (0.89-4.31) |
| 2018 | Castelló | case-control study | Spain | NA | 20-85 | stomach | 6258 | 3161 | Female | Mediterranean diet | NA | 1.80 (0.83-3.90) |
| 2019 | Bahrami | case-control study | Iran | NA | 30-79 | colorectum | 6258 | 3161 | Both male and female | Semi vegetarian | NA | 0.22 (0.14-0.37) |
| 2020 | Kim | case-control study | Korea | NA | 50 | stomach | 6258 | 3161 | Both male and female | Prudent diet | NA | 0.58 (0.41-0.84) |
| 2020 | Toorang | case-control study | Iran | NA | 40 | stomach | 6258 | 3161 | Both male and female | DASH diet | NA | 0.46 (0.26-0.83) |
| 2020 | Jafari Nasab | case-control study | Iran | NA | 58.19 | colorectum | 6258 | 3161 | Both male and female | Mediterranean diet | NA | 0.19 (0.09-0.38) |
| 2021 | Nojomi | cohort study | Iran | 10.1 | 40-75 | oesophagus | 6258 | 3161 | Both male and female | Mediterranean diet | 5.49 | 1.19 (1.05-1.35) |
| 2021 | Nojomi | cohort study | Iran | 10.1 | 40-75 | oesophagus | 6258 | 3161 | Both male and female | Mediterranean diet | 4.01 | 1.07 (0.95-1.19) |
| 2021 | Nojomi | cohort study | Iran | 10.1 | 40-75 | oesophagus | 6258 | 3161 | Both male and female | Mediterranean diet | 3.36 | 1.02 (0.90-1.17) |
| 2021 | Jafari Nasab | case-control study | Iran | NA | 58.19 | stomach | 6258 | 3161 | Both male and female | DASH diet | NA | 0.04 (0.01-0.11) |
| 2021 | Jafari Nasab | case-control study | Iran | NA | 58.19 | stomach | 6258 | 3161 | Female | DASH diet | NA | 0.07 (0.02-0.26) |
| 2021 | Jafari Nasab | case-control study | Iran | NA | 58.19 | stomach | 6258 | 3161 | Male | DASH diet | NA | 0.02 (0.00-0.13) |
| 2021 | Moussa | case-control study | USA | NA | 62.9 | liver | 6258 | 3161 | Both male and female | Lacto-vegetarian | T2 | 0.57 (0.40-0.83) |
| 2021 | Moussa | case-control study | USA | NA | 62.9 | liver | 6258 | 3161 | Both male and female | Lacto-vegetarian | T3 | 0.66 (0.46-0.94) |

***Abbreviations:*** USA: the United States of America, UK: the United Kingdom, NA: not available, DASH: dietary approaches to stop hypertension.

**eTable 4.2 Supplementary characteristics of the studies conducted on plant-based diets and cancer risk included in the meta-analysis.**

| **ID** | **Year** | **Author** | **Population** | **Dietary assessment** | **Dietary type** | **Dietary definition** | **Adjustment** |
| --- | --- | --- | --- | --- | --- | --- | --- |
| 1 | 1988 | Mills | Adventist | NA | Vegan | Eat only plant-based foods (no red meat poultry, fish, dairy, or eggs). | Age and sex. |
| 2 | 1994 | Centonze | Normal person | FFQ, 70 items | Vegan | Eat only plant-based foods (no red meat poultry, fish, dairy, or eggs). | Age, sex, level of education, smoking status, and modification of diet in the past. |
| 3 | 1998 | Slattery | Normal person | NA | Prudent diet | The prudent pattern was characterized by high intakes of vegetables, fruit, noodle, potatoes, soy products, mushroom, and seaweed. | Age, body mass index, long term physical activity, and total energy intake, those given for the total sample. |
| 4 | 2001 | Terry | Normal person | FFQ, 67 items | Semi vegetarian | Eat dairy, eggs and some red meat, poultry, and fish >= 1time/month but <1time/week. | Age, energy intake, body mass index, and education. |
| 5 | 2003 | Masaki | Normal person | FFQ，33items | Vegan | Eat only plant-based foods (no red meat poultry, fish, dairy, or eggs). | Age, BMI, education, history of peptic ulcer, family history of stomach cancer, status of cigarette smoking and alcohol drinking. |
| 6 | 2004 | Wu | Normal person | FFQ, 131 items | Prudent diet | The prudent pattern was characterized by high intakes of vegetables, fruit, noodle, potatoes, soy products, mushroom, and seaweed. | Age, family history of colorectal cancer, history of endoscopy, physical activity, pack-years of smoking before age 30, race, aspirin use, total energy intake and body mass index. |
| 7 | 2005 | Kim | Normal person | FFQ | Pesco-vegetarian | Eat fish, but no red meat, poultry, dairy, or eggs. | Age, body mass index, study area, energy intake, education level, physical activity, family history of colorectal cancer, smoking status and alcohol consumption. |
| 8 | 2005 | Nkondjock | Normal person | FFQ, 69 items | Vegan | Eat only plant-based foods (no red meat poultry, fish, dairy, or eggs). | Age, smoking, BMI, physical activity, province, educational attainment, and total energy intake. |
| 9 | 2005 | Rouillier | Normal person | FFQ | Vegan | Eat only plant-based foods (no red meat poultry, fish, dairy, or eggs). | Age, gender, energy, body mass index, exercise, tobacco, and alcohol. |
| 10 | 2008 | Campbell | Normal person | FFQ, 69 items | Prudent diet | The prudent pattern was characterized by high intakes of vegetables, fruit, noodle, potatoes, soy products, mushroom, and seaweed. | Age, strenuous physical activity 2 years prior, body mass index, education, province of residence, pack-years of smoking, and total energy intake. |
| 11 | 2009 | Wirfalt | Normal person | FFQ, 181 items | Semi vegetarian | Eat dairy, eggs and some red meat, poultry, and fish >= 1time/month but <1time/week. | Age, BMI, education, ethnicity, smoking, leisure time physical activity and total energy. In women also adjusted for hormone replacement therapy. |
| 12 | 2009 | Joshi | Normal person | NA | Vegan | Eat only plant-based foods (no red meat poultry, fish, dairy, or eggs). | NA. |
| 13 | 2010 | Kurotani | Normal person | FFQ, 211 items | Prudent diet | The prudent pattern was characterized by high intakes of vegetables, fruit, noodle, potatoes, soy products, mushroom, and seaweed. | Smoking, alcohol use, BMI 10 years earlier, type of job, leisure-time physical activity, parental colorectal cancer and energy intake. |
| 14 | 2010 | Buckland | Normal person | Mediterranean  diet score | Mediterranean diet | Mediterranean diet is characterized by high consumption of fruit, vegetables, cereals, legumes, nuts and seeds, fish and seafood, olive oil as the main fat source, moderate alcohol consumption (particularly red wine), a relatively low consumption of dairy products (mainly cheese and yoghurt), and red and processed meat. | Sex, BMI, educational level, smoking status, cigarette smoking intensity, and total energy intake. |
| 15 | 2010 | Miller | Normal person | FFQ, 137 items | Vegan | Eat only plant-based foods (no red meat poultry, fish, dairy, or eggs). | Age, BMI, education, energy intake, family history of colorectal cancer, postmenopausal hormone use, NSAID use, physical activity, and smoking. |
| 16 | 2011 | Magalhaes | Normal person | FFQ, 82 items | Semi vegetarian | Eat dairy, eggs and some red meat, poultry, and fish >= 1time/month but <1time/week. | Sex, age, education level, total energy intake, and physical activity. |
| 17 | 2011 | Reedy | Normal person | FFQ, 124 items | Vegan | Eat only plant-based foods (no red meat poultry, fish, dairy, or eggs). | Age, ethnicity, education, body mass index, smoking, physical activity, energy, and menopausal hormone therapy (women only) |
| 18 | 2012 | Bradshaw | Normal person | NA | Semi vegetarian | Eat dairy, eggs and some red meat, poultry, and fish >= 1time/month but <1time/week. | Matching strata (gender, race, age), total energy intake, tobacco use, beer consumption, liquor consumption, and education. |
| 19 | 2012 | Ibiebele | Normal person | FFQ, 139 items | Vegan | Eat only plant-based foods (no red meat poultry, fish, dairy, or eggs). | Age, sex, education, BMI 1 year ago, frequency of heartburn or acid reflux 10 years before diagnosis/recruitment, pack-years of smoking, non-steroidal anti-inflammatory drugs use, and total energy. |
| 20 | 2013 | Praud | Normal person | FFQ | Mediterranean diet | Mediterranean diet is characterized by high consumption of fruit, vegetables, cereals, legumes, nuts and seeds, fish and seafood, olive oil as the main fat source, moderate alcohol consumption (particularly red wine), a relatively low consumption of dairy products (mainly cheese and yoghurt), and red and processed meat. | Age, sex, study, year of interview, education, body mass index, tobacco smoking, family history, and total energy intake. |
| 21 | 2013 | Safar | Normal person | FFQ, 125 items | Pesco-laco-ovo-vegetarian | Eat fish, dairy, and eggs but no red meat or poultry. | Family history of CRC in first and second-degree relative, vegetable preparation, aspirin, acetaminophen, mineral and energy intake. |
| 22 | 2013 | Kontou | Normal person | FFQ, 69 items | Mediterranean diet | Mediterranean diet is characterized by high consumption of fruit, vegetables, cereals, legumes, nuts and seeds, fish and seafood, olive oil as the main fat source, moderate alcohol consumption (particularly red wine), a relatively low consumption of dairy products (mainly cheese and yoghurt), and red and processed meat. | Age, sex, and family history of CRC. |
| 23 | 2013 | Chan | Normal person | FFQ, 35 items | Prudent diet | The prudent pattern was characterized by high intakes of vegetables, fruit, noodle, potatoes, soy products, mushroom, and seaweed. | Age, race, education, diabetes, body mass index, smoking, alcohol drinking, physical activity, and total energy. |
| 24 | 2013 | Bosetti | Normal person | FFQ, 78 items | Mediterranean diet | Mediterranean diet is characterized by high consumption of fruit, vegetables, cereals, legumes, nuts and seeds, fish and seafood, olive oil as the main fat source, moderate alcohol consumption (particularly red wine), a relatively low consumption of dairy products (mainly cheese and yoghurt), and red and processed meat. | Centre, age, sex, year of interview, education, body mass index, tobacco smoking, alcohol consumption, history of diabetes, and total energy intake. |
| 25 | 2013 | Arem | Normal person | FFQ, 124 items | Semi vegetarian | Eat dairy, eggs and some red meat, poultry, and fish >= 1time/month but <1 time/week. | Daily caloric intake, sex, diabetes, body mass index and smoking status. |
| 26 | 2013 | Agnoli | Normal person | FFQ | Mediterranean diet | Mediterranean diet is characterized by high consumption of fruit, vegetables, cereals, legumes, nuts and seeds, fish and seafood, olive oil as the main fat source, moderate alcohol consumption (particularly red wine), a relatively low consumption of dairy products (mainly cheese and yoghurt), and red and processed meat. | Non-alcoholic energy intake, gender, age, BMI, smoking, education, and total physical activity; stratified for center. |
| 27 | 2013 | Bosetti2 | Normal person | NA | Vegan | Eat only plant-based foods (no red meat poultry, fish, dairy, or eggs). | Year of interview, education, body mass index, tobacco smoking, alcohol drinking, and diabetes. |
| 28 | 2013 | Zhang | Normal person | FFQ | Vegan | Eat only plant-based foods (no red meat poultry, fish, dairy, or eggs). | Age, body mass index, total energy intake, family income level, education level, family history of liver cancer in first-degree relatives, history of chronic viral hepatitis, history of chronic liver disease or cirrhosis, history of diabetes, history of cholelithiasis or cholecystectomy, vitamin C and E and multivitamin supplement use, and mutual adjustment for three dietary patterns. |
| 29 | 2014 | Kumagai | Normal person | FFQ, 40 items | Vegan | Eat only plant-based foods (no red meat poultry, fish, dairy, or eggs). | Age, sex, body mass index, smoking status, walking duration, education, total energy intake, and family history of colorectal cancer. |
| 30 | 2014 | Grosso | Normal person | FFQ | Mediterranean diet | Mediterranean diet is characterized by high consumption of fruit, vegetables, cereals, legumes, nuts and seeds, fish and seafood, olive oil as the main fat source, moderate alcohol consumption (particularly red wine), a relatively low consumption of dairy products (mainly cheese and yoghurt), and red and processed meat. | Age and sex and all the other variable significant at univariate analysis. |
| 31 | 2014 | Denova-Gutiérrez | Normal person | FFQ | Semi vegetarian | Eat dairy, eggs and some red meat, poultry, and fish >= 1time/month but <1 time/week. | Age, gender, education, cigarettes, energy intake, and H. pylori infection. |
| 32 | 2014 | Filomeno | Normal person | FFQ | Mediterranean diet | Mediterranean diet is characterized by high consumption of fruit, vegetables, cereals, legumes, nuts and seeds, fish and seafood, olive oil as the main fat source, moderate alcohol consumption (particularly red wine), a relatively low consumption of dairy products (mainly cheese and yoghurt), and red and processed meat. | Sex, quinquennia of age, study center, year of interview, education, tobacco smoking, body mass index, and total energy intake. |
| 33 | 2015 | Orlich | Seventh-Day Adventist | FFQ, 200 items | Vegan | Eat only plant-based foods (no red meat poultry, fish, dairy, or eggs). | Age, sex, race, educational level, moderate or vigorous exercise, smoking, alcohol, family history of colorectal cancer, history of peptic ulcer, history of inflammatory bowel disease, treatment for diabetes mellitus within the past year, aspirin use, statin therapy, prior colonoscopy or flexible sigmoidoscopy, supplemental calcium consumption, supplemental vitamin D, dietary energy, hormone therapy among menopausal women, BMI, fiber intake. |
| 34 | 2015 | Gilsing | Normal person | FFQ, 150 items | Lacto-ovo-vegetarian | Eat dairy, but no red meat, poultry, fish, or eggs. | Age, sex, total energy intake, cigarette smoking, alcohol consumption, BMI, no occupational physical activity and level of education. |
| 35 | 2015 | McLean | Normal person | FFQ, 106 items | Prudent diet | The prudent pattern was characterized by high intakes of vegetables, fruit, noodle, potatoes, soy products, mushroom, and seaweed. | 10-year age group, province, education, body mass index, pack-year smoking, moderate and strenuous activity, calcium supplementation and age at first pregnancy for women. |
| 36 | 2015 | Azizi | Normal person | FFQ, 123 items | Pesco-laco-ovo-vegetarian | Eat fish, dairy, and eggs but no red meat or poultry. | History of diabetes, family history of CRC in first-degree relative, physical activity and other confounding factors. |
| 37 | 2015 | Chen | Normal person | FFQ, 169 items | Vegan | Eat only plant-based foods (no red meat poultry, fish, dairy, or eggs). | Age, total energy intake, body mass index, marital status, education attainment, household income status, use of alcohol/tobacco/non-steroid anti-inflammatory drug (NSAID), family history of CRC, history of diabetes/colon screening procedure/ high cholesterol, reported hormone replacement therapy (females only), multivitamin supplements, and physical activities. |
| 38 | 2016 | Park | Normal person | FFQ, 106 items | Prudent diet | The prudent pattern was characterized by high intakes of vegetables, fruit, noodle, potatoes, soy products, mushroom, and seaweed. | Marital status, educational level, monthly income, BMI, occupation, smoking status, alcohol consumption, physical activity, first-degree family of colorectal cancer, and total energy intake. |
| 39 | 2017 | Jones | Normal person | FFQ, 217 items | Mediterranean diet | Mediterranean diet is characterized by high consumption of fruit, vegetables, cereals, legumes, nuts and seeds, fish and seafood, olive oil as the main fat source, moderate alcohol consumption (particularly red wine), a relatively low consumption of dairy products (mainly cheese and yoghurt), and red and processed meat. | Age, BMI, energy intake, physical activity, smoking status, socioeconomic status, and family history of colorectal cancer. |
| 40 | 2018 | Rada-Fernandez de | Normal person | FFQ, 217 items | Semi vegetarian | Eat dairy, eggs and some red meat, poultry, and fish >= 1 time/month but <1 time/week. | Age, body mass index, energy intake, physical activity, smoking status, family history of CRC in a first degree relative and socio-economic status. |
| 41 | 2018 | Shin | Normal person | FFQ, 138 items | Prudent diet | The prudent pattern was characterized by high intakes of vegetables, fruit, noodle, potatoes, soy products, mushroom, and seaweed. | Age, public health center area, BMI, smoking status, total physical activity, and log-transformed total energy intake. |
| 42 | 2018 | Castelló | Normal person | FFQ, 154 items | Prudent diet | The prudent pattern was characterized by high intakes of vegetables, fruit, noodle, potatoes, soy products, mushroom, and seaweed. | Sex, age, education, BMI, family history of gastric cancer, physical activity (METs), smoking status, caloric intake, and alcohol intake as fixed effects and province of residence as a random effect; percentages might not add up to 100 because of rounding. |
| 43 | 2019 | Bahrami | Normal person | FFQ, 147 items | Semi vegetarian | Eat dairy, eggs and some red meat, poultry, and fish >= 1 time/month but <1 time/week. | Age, cancer family history, CRC family history, physical activity, and calcium supplement use. |
| 44 | 2020 | Kim | Normal person | FFQ, 106 items | Prudent diet | The prudent pattern was characterized by high intakes of vegetables, fruit, noodle, potatoes, soy products, mushroom, and seaweed. | Age, sex, total energy intake, family history of GC, smoking status, regular exercise, education, occupation, monthly income, the other dietary pattern, and H. pylori infection. |
| 45 | 2020 | Toorang | Normal person | NA | DASH diet | Dietary approaches to stop hypertension, it emphasizes high intake of fruit, vegetable, nuts, legumes, low fat dairy products, and whole grains and recommends low intake of sodium, sweetened beverages, and red and processed meats. | Age, sex, energy intake, education, marital status, residential place, alcohol intake, smoking status, Hpylori infection, BMI. |
| 46 | 2020 | Jafari Nasab | Normal person | NA | Mediterranean diet | Mediterranean diet is characterized by high consumption of fruit, vegetables, cereals, legumes, nuts and seeds, fish and seafood, olive oil as the main fat source, moderate alcohol consumption (particularly red wine), a relatively low consumption of dairy products (mainly cheese and yoghurt), and red and processed meat. | Age, comorbidities, cancer family history, common ways of cooking, level of salt intake, physical activity, and calcium supplement use. |
| 47 | 2021 | Nojomi | Normal person | FFQ, 30 items | Mediterranean diet | Mediterranean diet is characterized by high consumption of fruit, vegetables, cereals, legumes, nuts and seeds, fish and seafood, olive oil as the main fat source, moderate alcohol consumption (particularly red wine), a relatively low consumption of dairy products (mainly cheese and yoghurt), and red and processed meat. | Age, sex, total energy, place of residence, smoking, wealth score, ethnicity, opiate use, BMI, education, marital status, and physical activity score. |
| 48 | 2021 | Jafari Nasab | Normal person | NA | DASH diet | Dietary approaches to stop hypertension, it emphasizes high intake of fruit, vegetable, nuts, legumes, low fat dairy products, and whole grains and recommends low intake of sodium, sweetened beverages, and red and processed meats. | BMI, age, alcohol consumption, physical activity, history of diabetes, CHD and hypertension, family history of cancer. |
| 49 | 2021 | Moussa | Normal person | FFQ, 35 items | Lacto-vegetarian | Eat dairy and eggs but no red meat, poultry, and fish. | Sex, age, race, history of type 2 diabetes mellitus, body mass index, and hepatitis B or C virus infection. |

***Abbreviations:*** HRT: Hormone replacement therapy, BMI: Body mass index, OCP: oral contraceptive pill, IPAQ score: International physical activity questionnaire, CRC: Colorectal cancer, FFQ: food frequency questionnaire, NA: not available, DASH: dietary approaches to stop hypertension.

**eTable 5. Food composition of plant-based diets.**

| **Year** | **Author** | **Whole grains** | **Fruits** | **Vegetables** | **Nuts** | **Legumes** | **Vegetable oil** | **Tea & Coffee** | **Fruit juices** | **Potatoes** | **Sugar sweetened beverages** | **Sweets & desserts** | **Animal Fat** | **Dairy** | **Eggs** | **Fish & seafood** | **Poultry** | **Red meat** |
| --- | --- | --- | --- | --- | --- | --- | --- | --- | --- | --- | --- | --- | --- | --- | --- | --- | --- | --- |
| **1988** | **Mills** | × | √ | √ | x | √ | x | x | x | x | x | x | x | x | √ | √ | √ | √ |
| **1994** | **Centonze** | × | √ | √ | × | × | × | √ | × | √ | √ | × | × | √ | √ | √ | × | √ |
| **1998** | **Slattery** | √ | √ | √ | √ | √ | × | √ | √ | √ | √ | × | × | √ | √ | √ | √ | √ |
| **2001** | **Terry** | √ | √ | √ | x | x | x | √ | √ | √ | x | √ | x | √ | √ | √ | √ | √ |
| **2003** | **Masaki** | x | √ | √ | x | x | x | √ | √ | x | x | √ | x | √ | √ | √ | √ | √ |
| **2004** | **Wu** | x | √ | √ | √ | x | x | √ | x | √ | x | √ | x | x | √ | √ | √ | √ |
| **2005** | **Kim** | x | √ | √ | x | √ | x | √ | √ | √ | x | √ | x | √ | √ | √ | √ | √ |
| **2005** | **Rouillier** | x | √ | √ | x | x | x | √ | √ | x | x | x | x | x | √ | √ | x | √ |
| **2006** | **Nkondjock** | √ | √ | √ | √ | √ | x | √ | √ | √ | x | x | x | √ | √ | √ | √ | √ |
| **2008** | **Campbell** | x | √ | √ | x | x | x | √ | x | √ | x | x | x | x | x | x | x | x |
| **2009** | **Joshi** | × | √ | √ | × | × | × | √ | × | × | × | × | × | √ | √ | √ | √ | √ |
| **2009** | **Wirfalt** | NA | NA | NA | NA | NA | NA | NA | NA | NA | NA | NA | NA | NA | NA | NA | NA | NA |
| **2010** | **Kurotani** | x | √ | √ | √ | √ | x | x | x | √ | x | x | x | √ | √ | √ | √ | √ |
| **2010** | **Buckland** | x | √ | √ | x | √ | x | x | x | x | x | x | x | √ | x | √ | x | √ |
| **2010** | **Miller** | √ | √ | √ | √ | √ | x | x | x | √ | x | √ | x | x | x | √ | √ | √ |
| **2011** | **Magalha˜es** | x | √ | √ | √ | √ | √ | √ | x | √ | √ | √ | x | √ | √ | √ | x | √ |
| **2011** | **Reedy** | NA | NA | NA | NA | NA | NA | NA | NA | NA | NA | NA | NA | NA | NA | NA | NA | NA |
| **2012** | **Bradshaw** | √ | √ | √ | x | √ | x | x | x | √ | x | √ | x | x | x | x | √ | √ |
| **2012** | **Ibiebele** | x | √ | √ | √ | √ | x | √ | √ | √ | x | √ | x | √ | √ | √ | √ | √ |
| **2013** | **Praud** | x | √ | √ | x | √ | x | x | x | √ | x | x | x | √ | x | √ | x | √ |
| **2013** | **Safari** | √ | √ | √ | √ | √ | x | √ | x | x | x | √ | x | √ | x | √ | x | √ |
| **2013** | **Kontou** | x | √ | √ | √ | √ | x | x | x | √ | x | x | x | √ | x | √ | √ | √ |
| **2013** | **Chan** | √ | √ | √ | x | √ | x | √ | √ | √ | x | x | x | x | √ | √ | √ | x |
| **2013** | **Bosetti** | NA | NA | NA | NA | NA | NA | NA | NA | NA | NA | NA | NA | NA | NA | NA | NA | NA |
| **2013** | **Arem** | √ | √ | √ | x | √ | x | x | √ | x | x | x | x | x | x | x | x | x |
| **2013** | **Agnoli** | x | √ | √ | √ | √ | x | x | x | √ | x | x | x | x | x | √ | x | √ |
| **2013** | **Bosetti2** | x | √ | √ | x | √ | x | √ | √ | √ | x | √ | x | √ | √ | √ | x | √ |
| **2013** | **Zhang** | NA | NA | NA | NA | NA | NA | NA | NA | NA | NA | NA | NA | NA | NA | NA | NA | NA |
| **2014** | **Kumagai** | x | √ | √ | x | x | x | x | x | x | x | x | x | √ | x | x | x | x |
| **2014** | **Grosso** | x | √ | √ | x | √ | x | x | x | √ | x | x | x | √ | x | √ | √ | √ |
| **2014** | **Denova** | √ | √ | √ | x | x | x | √ | √ | √ | x | √ | x | x | √ | √ | x | x |
| **2014** | **Filomeno** | x | √ | √ | x | √ | x | x | x | √ | x | x | x | x | x | √ | x | √ |
| **2015** | **McLean** | √ | √ | √ | √ | √ | x | x | x | x | x | x | x | √ | x | x | x | √ |
| **2015** | **Azizi** | x | √ | √ | x | x | x | x | x | x | x | x | x | √ | x | √ | x | x |
| **2015** | **Azizi** | x | √ | √ | √ | x | √ | √ | x | x | x | √ | x | √ | x | √ | √ | √ |
| **2015** | **Orlich** | NA | NA | NA | NA | NA | NA | NA | NA | NA | NA | NA | NA | NA | NA | NA | NA | NA |
| **2015** | **Gilsing** | x | √ | √ | x | √ | x | x | x | x | x | x | x | √ | √ | √ | √ | √ |
| **2016** | **Park** | x | √ | x | x | √ | x | x | x | x | x | √ | x | x | √ | √ | √ | √ |
| **2017** | **Jones** | x | √ | √ | √ | √ | x | x | x | x | x | x | x | √ | x | √ | x | √ |
| **2018** | **Shin** | x | √ | √ | x | √ | x | x | √ | √ | x | x | x | x | x | x | x | x |
| **2018** | **Castelló** | √ | √ | √ | √ | √ | x | x | √ | √ | x | √ | x | √ | √ | √ | x | √ |
| **2018** | **Jauregui** | x | √ | √ | x | x | x | x | x | x | x | x | x | x | x | x | √ | √ |
| **2019** | **Kim** | x | √ | √ | √ | √ | x | √ | √ | x | x | √ | x | √ | √ | √ | √ | √ |
| **2019** | **Bahra** | x | √ | √ | √ | √ | x | √ | x | x | x | x | x | x | √ | x | √ | x |
| **2020** | **Toorang** | x | √ | √ | √ | √ | x | x | x | x | √ | x | √ | √ | x | x | x | x |
| **2020** | **JafariNasab** | √ | √ | √ | √ | √ | √ | x | x | x | x | √ | x | √ | √ | √ | √ | √ |
| **2021** | **Nojomi** | x | √ | x | x | √ | x | √ | x | √ | x | x | x | √ | √ | √ | √ | √ |
| **2021** | **JafariNasab** | √ | √ | √ | √ | √ | x | x | x | x | √ | x | x | √ | x | x | x | √ |
| **2021** | **Moussa** | √ | √ | √ | √ | √ | x | √ | √ | √ | x | √ | x | √ | √ | √ | √ | √ |

***Abbreviation:*** NA: not available.

**etable 6.1 Risk of Bias in Non-randomized Studies of Interventions (ROBINS-I):1988-2012**

| **Author** | Mills | Centonze | Slattery | Terry | Masaki | Wu | Kim | Nkondjock | Rouillier | Campbell | Wirfalt | Joshi | Kurotani | Buckland | Miller | Magalhaes | Reedy | Bradshaw | Ibiebele |
| --- | --- | --- | --- | --- | --- | --- | --- | --- | --- | --- | --- | --- | --- | --- | --- | --- | --- | --- | --- |
| **Year** | 1988 | 1994 | 1998 | 2001 | 2003 | 2004 | 2005 | 2005 | 2005 | 2008 | 2009 | 2009 | 2010 | 2010 | 2010 | 2011 | 2011 | 2012 | 2012 |
| **1.Bias due to confounding** | | | | | | | | | | | | | | | | | | | |
| **1.1 Is there potential for confounding of the effect of intervention in this study?** | Yes | Yes | Yes | Yes | Yes | Yes | Yes | Yes | Yes | Yes | Yes | Yes | Yes | Yes | Yes | Yes | Yes | Yes | Yes |
| **1.2 Was the analysis based on splitting participants' follow-up time according to intervention received?** | No | No | No | No | No | No | PN | NI | No | NI | PN | NI | NI | PN | NI | No | PN | NI | NI |
| **1.3 Were intervention discontinuations or switches likely to be related to factors that are prognostic for the outcome?** | NI | No | No | No | No | NI | No | NI | NI | NI | NI | NI | NI | NI | NI | NI | NI | NI | NI |
| **1.4 Did the authors use an appropriate analysis method that controlled for all the important confounding domains?** | Yes | Yes | NI | Yes | Yes | Yes | Yes | Yes | Yes | Yes | Yes | No | Yes | Yes | Yes | Yes | Yes | Yes | Yes |
| **1.5 Were confounding domains that were controlled for measured validly and reliably by the variables available in this study?** | PY | Yes | PY | Yes | Yes | Yes | Yes | PY | Yes | Yes | Yes | NI | Yes | Yes | Yes | Yes | Yes | Yes | Yes |
| **1.6 Did the authors control for any post-intervention variables that could have been affected by the intervention?** | Yes | Yes | NI | PY | PY | PY | PY | PY | Yes | Yes | PY | NI | NI | PY | NI | PY | PY | PY | PY |
| **1.7 Did the authors use an appropriate analysis method that controlled for all the important confounding domains and for time-varying confounding?** | PY | Yes | PN | Yes | NI | Yes | PY | PY | NI | NI | PY | NI | NI | Yes | NI | NI | PY | Yes | PN |
| **1.8 Were confounding domains that were controlled for measured validly and reliably by the variables available in this study?** | Yes | Yes | NI | Yes | Yes | Yes | Yes | PY | PY | PY | Yes | NI | NI | Yes | PN | NI | PY | Yes | PY |
| **Risk of bias judgment** | Moderate | Low | Serious | Low | Low | Low | Moderate | Moderate | Moderate | Moderate | Low | Serious | Moderate | Low | Moderate | Moderate | Low | Moderate | Moderate |
| **What is the predicted direction of bias due to confounding?** | FE | FE | Unpredictable | FE | FE | FE | FE | FE | FE | FE | FE | Unpredictable | FE | FE | FE | FE | FE | FE | FE |
| **2.Bias in selection of participants into the study** | | | | | | | | | | | | | | | | | | | |
| **2.1Was selection of participants into the study (or into the analysis) based on participant characteristics observed after the start of intervention?** | No | Yes | Yes | No | No | No | No | Yes | Yes | Yes | No | Yes | Yes | No | Yes | Yes | No | Yes | Yes |
| **2.2 Were the post-intervention variables that influenced selection likely to be associated with intervention?** | **_** | No | No | **_** | **_** | **_** | _ | No | No | No | _ | No | No | _ | No | No | _ | No | No |
| **2.3 Were the post-intervention variables that influenced selection likely to be influenced by the outcome or a cause of the outcome?** | **_** | Yes | Yes | _ | _ | **_** | _ | Yes | Yes | Yes | _ | Yes | Yes | _ | Yes | Yes | _ | Yes | Yes |
| **2.4 Do start of follow-up and start of intervention coincide for most participants?** | PY | _ | _ | Yes | Yes | Yes | Yes | _ | _ | _ | Yes | _ | _ | No | _ | _ | Yes | _ | _ |
| **2.5. Were adjustment techniques used that are likely to correct for the presence of selection bias?** | No | _ | _ | PN | No | No | No | _ | _ | _ | No | _ | _ | Yes | _ | _ | PN | _ | _ |
| **Risk of bias judgment** | Low | Moderate | Moderate | Moderate | Moderate | Low | Low | Moderate | Moderate | Moderate | Low | Moderate | Moderate | Low | Moderate | Moderate | Low | Moderate | Moderate |
|  |  |  |  |  |  |  |  |  |  |  |  |  |  |  |  |  |  |  |  |
| **What is the predicted direction of bias due to confounding?** | Towards null | Towards null | Towards null | Towards null | FE | FE | FE | FE | FE | FE | FE | FE | FE | FE | FE | FE | FE | FE | FE |
| **3.Bias in classification of interventions** | | | | | | | | | | | | | | | | | | | |
| **3.1 Were intervention groups clearly defined?** | Yes | Yes | Yes | Yes | Yes | Yes | Yes | Yes | Yes | Yes | Yes | Yes | Yes | Yes | Yes | Yes | Yes | Yes | Yes |
| **3.2 Was the information used to define intervention groups recorded at the start of the intervention?** | Yes | No | No | Yes | Yes | Yes | Yes | No | Yes | No | Yes | No | No | No | No | No | Yes | PN | No |
| **3.3 Could classification of intervention status have been affected by knowledge of the outcome or risk of the outcome?** | No | Yes | Yes | No | No | Yes | No | Yes | Yes | Yes | No | Yes | Yes | No | Yes | Yes | PN | Yes | Yes |
| **Risk of bias judgment** | Low | Moderate | Moderate | Low | Low | Moderate | Low | Moderate | Moderate | Moderate | Low | Moderate | Moderate | Low | Moderate | Moderate | Low | Moderate | Moderate |
| **What is the predicted direction of bias due to confounding?** | FE | FE | FE | FE | FE | FE | FE | FE | FE | FE | FE | FE | FE | FE | FE | FE | FE | FE | FE |
| **4.Bias due to deviations from intended interventions** | | | | | | | | | | | | | | | | | | | |
| **If your aim for this study is to assess the effect of: 1) assignment to intervention, answer questions 4.1 and 4.2; 2) starting and adhering to intervention, answer questions 4.3 to 4.6** | | | | | | | | | | | | | | | | | | | |
| **4.1 Were there deviations from the intended intervention beyond what would be expected in usual practice?** | Yes | NI | No | No | NI | NI | NI | PN | NI | PN | NI | NI | PN | No | PN | PN | NI | NI | NI |
| **4.2 Were these deviations from intended intervention unbalanced between groups and likely to have affected the outcome?** | No | No | No | NI | PN | NI | NI | No | NI | No | No | NI | PN | No | No | No | NI | NI | NI |
| **4.3 Were important co-interventions balanced across intervention groups?** | _ | _ | _ | _ | _ | _ | _ | _ | _ | _ | _ | _ | _ | _ | _ | _ | _ | _ | _ |
| **4.4 Was the intervention implemented successfully for most participants?** | _ | _ | _ | _ | _ | _ | _ | _ | _ | _ | _ | _ | _ | _ | _ | _ | _ | _ | _ |
| **4.5 Did study participants adhere to the assigned intervention regimen?** | _ | _ | _ | _ | _ | _ | _ | _ | _ | _ | _ | _ | _ | _ | _ | _ | _ | _ | _ |
| **4.6 Was an appropriate analysis used to estimate the effect of starting and adhering to the intervention?** | _ | _ | _ | _ | _ | _ | _ | _ | _ | _ | _ | _ | _ | _ | _ | _ | _ | _ | _ |
| **Risk of bias judgment** | Low | Moderate | Low | Low | Low | NI | NI | Low | NI | Low | NI | NI | Low | Low | Low | Low | NI | NI | NI |
| **What is the predicted direction of bias due to confounding?** | FE | FE | FE | FE | FE | Unpredictable | Unpredictable | FE | Unpredictable | FE | Unpredictable | Unpredictable | FE | Towards null | FE | FE | Unpredictable | Unpredictable | Unpredictable |
| **5.Bias due to missing data** | | | | | | | | | | | | | | | | | | | |
| **5.1 Were outcome data available for all, or nearly all, participants?** | Yes | Yes | Yes | Yes | Yes | Yes | Yes | Yes | Yes | Yes | Yes | Yes | Yes | Yes | Yes | Yes | Yes | Yes | Yes |
| **5.2 Were participants excluded due to missing data on intervention status?** | Yes | Yes | Yes | Yes | Yes | Yes | Yes | Yes | Yes | Yes | Yes | Yes | Yes | Yes | Yes | Yes | Yes | Yes | Yes |
| **5.3 Were participants excluded due to missing data on other variables needed for the analysis?** | NI | Yes | Yes | Yes | Yes | Yes | PN | Yes | Yes | Yes | Yes | PY | Yes | Yes | NI | Yes | Yes | Yes | Yes |
| **5.4 Are the proportion of participants and reasons for missing data similar across interventions?** | NI | PY | Yes | PY | PY | PY | PY | PN | PN | PY | NI | Yes | PY | PY | NI | NI | NI | PN | NI |
| **5.5 Is there evidence that results were robust to the presence of missing data?** | No | No | No | No | No | No | No | No | No | No | No | No | No | No | No | No | No | No | No |
| **Risk of bias judgment** | Moderate | Moderate | Moderate | Low | Moderate | Moderate | Moderate | Moderate | Moderate | Moderate | Moderate | Low | Low | Low | Moderate | Moderate | Moderate | Low | Moderate |
| **What is the predicted direction of bias due to confounding?** | Towards null | FE | FE | FE | FE | FE | FE | FE | FE | FE | FE | FE | FE | FE | Towards null | Towards null | Towards null | FE | Towards null |
| **6.Bias in measurement of outcomes** | | | | | | | | | | | | | | | | | | | |
| **6.1 Could the outcome measure have been influenced by knowledge of the intervention received?** | No | No | No | No | No | No | No | No | No | No | No | No | No | No | No | No | No | No | No |
| **6.2 Were outcome assessors aware of the intervention received by study participants?** | Yes | Yes | Yes | Yes | Yes | Yes | Yes | Yes | Yes | Yes | Yes | Yes | Yes | Yes | Yes | Yes | Yes | Yes | Yes |
| **6.3 Were the methods of outcome assessment comparable across intervention groups?** | Yes | Yes | Yes | Yes | Yes | Yes | Yes | Yes | Yes | Yes | Yes | Yes | Yes | Yes | Yes | Yes | Yes | Yes | Yes |
| **6.4 Were any systematic errors in measurement of the outcome related to intervention received?** | No | No | No | No | No | No | No | No | No | No | No | No | No | No | No | No | No | No | No |
| **Risk of bias judgment** | Moderate | Moderate | Moderate | Moderate | Moderate | Moderate | Moderate | Moderate | Moderate | Moderate | Moderate | Moderate | Moderate | Moderate | Moderate | Moderate | Moderate | Moderate | Moderate |
| **What is the predicted direction of bias due to confounding?** | FE | FE | FE | FE | FE | FE | FE | FE | FE | FE | FE | FE | FE | FE | FE | FE | FE | FE | FE |
| **7.Bias in selection of the reported result** | | | | | | | | | | | | | | | | | | | |
| **7.1 … multiple outcome measurements within the outcome domain?** | No | No | No | No | No | No | No | No | No | No | No | No | No | No | No | No | No | No | No |
| **7.2 ... multiple analyses of the intervention-outcome relationship?** | PN | PN | PN | PN | PN | PN | PN | PN | PN | PN | PN | PN | PN | PN | PN | PN | PN | PN | PN |
| **7.3 ... different subgroups?** | No | PN | PN | PN | PN | PN | PN | PN | PN | PN | PN | PN | PN | PN | PN | PN | PN | PN | PN |
| **Risk of bias judgment** | Low | Low | Low | Low | Low | Low | Low | Low | Low | Low | Low | Low | Low | Low | Low | Low | Low | Low | Low |
| **What is the predicted direction of bias due to confounding?** | FE | FE | FE | FE | FE | FE | FE | FE | FE | FE | FE | FE | FE | FE | FE | FE | FE | FE | FE |
| **Risk of bias judgment** | Moderate | Moderate | Moderate | Moderate | Moderate | Moderate | Moderate | Moderate | Moderate | Moderate | Moderate | Moderate | Moderate | Moderate | Moderate | Moderate | Moderate | Moderate | Moderate |
| **What is the predicted direction of bias due to confounding?** | FE | FE | FE | FE | FE | FE | FE | FE | FE | FE | FE | FE | FE | FE | FE | FE | FE | FE | FE |

***Abbreviations:*** PY: Probably Yes; PN: Probably No; NI: No Information; FE: Favours experimental.

**etable 6.2 Risk of Bias in Non-randomized Studies of Interventions (ROBINS-I): 2013-2017**

| **Author** | Praud | Safar | Kontou | Chan | Bosetti | Arem | Agnoli | Bosetti2 | Zhang | Kumagai | Grosso | Denova-Gutiérrez | Filomeno | Orlich | Gilsing | McLean | Azizi | Chen | Park | Jones |
| --- | --- | --- | --- | --- | --- | --- | --- | --- | --- | --- | --- | --- | --- | --- | --- | --- | --- | --- | --- | --- |
| **Year** | 2013 | 2013 | 2013 | 2013 | 2013 | 2013 | 2013 | 2013 | 2013 | 2014 | 2014 | 2014 | 2014 | 2015 | 2015 | 2015 | 2015 | 2015 | 2016 | 2017 |
| **1.Bias due to confounding** |  |  |  |  |  |  |  |  |  |  |  |  |  |  |  |  |  |  |  |  |
| **1.1 Is there potential for confounding of the effect of intervention in this study?** | Yes | Yes | Yes | Yes | Yes | Yes | Yes | Yes | Yes | Yes | Yes | Yes | Yes | Yes | Yes | Yes | Yes | Yes | Yes | Yes |
| **1.2 Was the analysis based on splitting participants' follow-up time according to intervention received?** | NI | NI | NI | NI | NI | NI | PN | NI | NI | PN | NI | NI | NI | PN | PN | NI | NI | NI | Yes | NI |
| **1.3 Were intervention discontinuations or switches likely to be related to factors that are prognostic for the outcome?** | NI | NI | NI | NI | NI | NI | NI | NI | NI | NI | NI | NI | NI | NI | NI | NI | NI | NI | NI | NI |
| **1.4 Did the authors use an appropriate analysis method that controlled for all the important confounding domains?** | Yes | Yes | Yes | Yes | Yes | Yes | Yes | Yes | Yes | Yes | PY | PY | PN | Yes | Yes | Yes | Yes | Yes | Yes | Yes |
| **1.5 Were confounding domains that were controlled for measured validly and reliably by the variables available in this study?** | Yes | Yes | Yes | Yes | Yes | Yes | Yes | Yes | Yes | Yes | PY | Yes | PN | Yes | Yes | Yes | Yes | Yes | Yes | Yes |
| **1.6 Did the authors control for any post-intervention variables that could have been affected by the intervention?** | NI | PY | PY | Yes | PN | Yes | PY | No | Yes | PY | PY | PY | PY | PY | PY | PY | PY | PY | PY | PY |
| **1.7 Did the authors use an appropriate analysis method that controlled for all the important confounding domains and for time-varying confounding?** | PY | PN | PN | PN | No | Yes | Yes | NI | Yes | Yes | Yes | Yes | PN | Yes | Yes | PY | Yes | PY | PY | PY |
| **1.8 Were confounding domains that were controlled for measured validly and reliably by the variables available in this study?** | Yes | PY | PY | Yes | Yes | Yes | Yes | Yes | Yes | Yes | Yes | Yes | NI | Yes | Yes | Yes | Yes | Yes | Yes | Yes |
| **Risk of bias judgment** | Low | Moderate | Moderate | Moderate | Low | Low | Low | Moderate | Low | Low | Low | Low | Moderate | Low | Low | Low | Low | Low | Low | Low |
| **What is the predicted direction of bias due to confounding?** | FE | FE | FE | FE | FE | FE | FE | FE | FE | FE | FE | FE | FE | FE | FE | FE | FE | FE | FE | FE |
| **2.Bias in selection of participants into the study** | | | | | | | | | | | | | | | | | | | | |
| **2.1Was selection of participants into the study (or into the analysis) based on participant characteristics observed after the start of intervention?** | Yes | Yes | Yes | Yes | Yes | No | No | Yes | No | No | Yes | Yes | Yes | No | No | Yes | Yes | Yes | Yes | No |
| **2.2 Were the post-intervention variables that influenced selection likely to be associated with intervention?** | No | No | No | No | No | _ | _ | No | _ | _ | No | No | No | _ | _ | No | No | No | No | _ |
| **2.3 Were the post-intervention variables that influenced selection likely to be influenced by the outcome or a cause of the outcome?** | Yes | Yes | Yes | Yes | Yes | _ | _ | Yes | _ | _ | Yes | Yes | Yes | _ | _ | Yes | Yes | Yes | Yes | _ |
| **2.4 Do start of follow-up and start of intervention coincide for most participants?** | **_** | _ | _ | _ | _ | Yes | Yes | _ | Yes | PY | _ | _ | _ | Yes | Yes | _ | _ | _ | _ | Yes |
| **2.5. Were adjustment techniques used that are likely to correct for the presence of selection bias?** | **_** | _ | _ | _ | _ | PN | PN | _ | PN | PN | _ | _ | _ | PN | PN | _ | _ | _ | _ | PN |
| **Risk of bias judgment** | Moderate | Moderate | Moderate | Moderate | Moderate | Low | Low | Moderate | Low | Low | Moderate | Moderate | Moderate | Low | Low | Moderate | Moderate | Moderate | Moderate | Low |
| **What is the predicted direction of bias due to confounding?** | FE | FE | FE | FE | FE | FE | FE | FE | FE | FE | FE | FE | FE | FE | FE | FE | FE | FE | FE | FE |
| **3.Bias in classification of interventions** |  |  |  |  |  |  |  |  |  |  |  |  |  |  |  |  |  |  |  |  |
| **3.1 Were intervention groups clearly defined?** | Yes | Yes | Yes | Yes | Yes | Yes | Yes | Yes | Yes | Yes | Yes | Yes | Yes | Yes | Yes | Yes | Yes | Yes | Yes | Yes |
| **3.2 Was the information used to define intervention groups recorded at the start of the intervention?** | PN | No | No | No | No | Yes | Yes | No | No | Yes | No | No | No | Yes | Yes | No | No | No | No | Yes |
| **3.3 Could classification of intervention status have been affected by knowledge of the outcome or risk of the outcome?** | Yes | Yes | Yes | Yes | Yes | No | No | Yes | No | No | Yes | Yes | Yes | No | No | Yes | Yes | Yes | Yes | No |
| **Risk of bias judgment** | Moderate | Moderate | Moderate | Moderate | Moderate | Low | Low | Moderate | Low | Low | Moderate | Moderate | Moderate | Low | Low | Moderate | Moderate | Moderate | Moderate | Low |
| **What is the predicted direction of bias due to confounding?** | FE | Towards null | Towards null | Towards null | FE | FE | FE | Towards null | FE | FE | Towards null | Towards null | Towards null | FE | FE | Towards null | Towards null | Towards null | Towards null | FE |
| **4.Bias due to deviations from intended interventions** | | | | | | | | | | | | | | | | | | | | |
| **If your aim for this study is to assess the effect of : 1) assignment to intervention, answer questions 4.1 and 4.2; 2) starting and adhering to intervention, answer questions 4.3 to 4.6** | | | | | | | | | | | | | | | | | | | | |
| **4.1 Were there deviations from the intended intervention beyond what would be expected in usual practice?** | NI | NI | NI | NI | NI | NI | NI | NI | NI | NI | PN | PN | PN | NI | NI | PN | PN | PN | PN | NI |
| **4.2 Were these deviations from intended intervention unbalanced between groups and likely to have affected the outcome?** | NI | NI | NI | NI | NI | NI | NI | NI | NI | NI | PN | PN | PN | NI | NI | PN | PN | PN | PN | NI |
| **4.3 Were important co-interventions balanced across intervention groups?** | **_** | **_** | **_** | **_** | **_** | **_** | **_** | **_** | **_** | **_** | **_** | **_** | **_** | **_** | **_** | **_** | **_** | **_** | **_** | **_** |
| **4.4 Was the intervention implemented successfully for most participants?** | **_** | **_** | **_** | **_** | **_** | **_** | **_** | **_** | **_** | **_** | **_** | **_** | **_** | **_** | **_** | **_** | **_** | **_** | **_** | **_** |
| **4.5 Did study participants adhere to the assigned intervention regimen?** | **_** | **_** | **_** | **_** | **_** | **_** | **_** | **_** | **_** | **_** | **_** | **_** | **_** | **_** | **_** | **_** | **_** | **_** | **_** | **_** |
| **4.6 Was an appropriate analysis used to estimate the effect of starting and adhering to the intervention?** | **_** | **_** | **_** | **_** | **_** | **_** | **_** | **_** | **_** | **_** | **_** | **_** | **_** | **_** | **_** | **_** | **_** | **_** | **_** | **_** |
| **Risk of bias judgment** | NI | NI | NI | NI | NI | NI | NI | NI | NI | NI | Low | Low | Low | NI | NI | Low | Low | Low | Low | NI |
| **What is the predicted direction of bias due to confounding?** | Unpredictable | Unpredictable | Unpredictable | Unpredictable | Unpredictable | Unpredictable | Unpredictable | Unpredictable | Unpredictable | Unpredictable | FE | FE | FE | Unpredictable | Unpredictable | FE | FE | FE | FE | Unpredictable |
| **5.Bias due to missing data** |  |  |  |  |  |  |  |  |  |  |  |  |  |  |  |  |  |  |  |  |
| **5.1 Were outcome data available for all, or nearly all, participants?** | Yes | Yes | Yes | Yes | Yes | Yes | Yes | Yes | Yes | Yes | Yes | PY | Yes | Yes | Yes | Yes | Yes | Yes | Yes | Yes |
| **5.2 Were participants excluded due to missing data on intervention status?** | Yes | Yes | Yes | Yes | Yes | Yes | Yes | Yes | Yes | Yes | NI | NI | NI | NI | NI | No | NI | Yes | PY | NI |
| **5.3 Were participants excluded due to missing data on other variables needed for the analysis?** | Yes | Yes | Yes | Yes | Yes | Yes | NI | NI | PY | Yes | NI | NI | NI | NI | NI | Yes | NI | Yes | PY | NI |
| **5.4 Are the proportion of participants and reasons for missing data similar across interventions?** | NI | NI | Yes | Yes | Yes | NI | NI | NI | Yes | PY | PY | PY | NI | NI | NI | PN | NI | PY | Yes | NI |
| **5.5 Is there evidence that results were robust to the presence of missing data?** | No | No | No | No | No | No | No | No | No | No | No | No | No | No | No | No | No | No | No | No |
| **Risk of bias judgment** | Moderate | Low | Low | Low | Low | Moderate | Low | Moderate | Low | Low | Moderate | Low | Moderate | Moderate | Moderate | Low | Moderate | Low | Low | Moderate |
| **What is the predicted direction of bias due to confounding?** | Towards null | FE | FE | FE | FE | Towards null | FE | Towards null | FE | FE | FE | FE | FE | FE | FE | FE | FE | FE | FE | FE |
| **6.Bias in measurement of outcomes** |  |  |  |  |  |  |  |  |  |  |  |  |  |  |  |  |  |  |  |  |
| **6.1 Could the outcome measure have been influenced by knowledge of the intervention received?** | No | No | No | No | No | No | No | No | No | No | No | No | No | No | No | No | No | No | No | No |
| **6.2 Were outcome assessors aware of the intervention received by study participants?** | Yes | Yes | Yes | Yes | Yes | Yes | Yes | Yes | Yes | Yes | Yes | Yes | Yes | Yes | Yes | Yes | Yes | Yes | Yes | Yes |
| **6.3 Were the methods of outcome assessment comparable across intervention groups?** | Yes | Yes | Yes | Yes | Yes | Yes | Yes | Yes | Yes | Yes | Yes | Yes | Yes | Yes | Yes | Yes | Yes | Yes | Yes | Yes |
| **6.4 Were any systematic errors in measurement of the outcome related to intervention received?** | No | No | No | No | No | No | No | No | No | No | No | No | No | No | No | No | No | No | No | No |
| **Risk of bias judgment** | Moderate | Moderate | Moderate | Moderate | Moderate | Moderate | Moderate | Moderate | Moderate | Moderate | Moderate | Moderate | Moderate | Moderate | Moderate | Moderate | Moderate | Moderate | Moderate | Moderate |
| **What is the predicted direction of bias due to confounding?** | FE | FE | FE | FE | FE | FE | FE | FE | FE | FE | FE | FE | FE | FE | FE | FE | FE | FE | FE | FE |
| **7.Bias in selection of the reported result** |  |  |  |  |  |  |  |  |  |  |  |  |  |  |  |  |  |  |  |  |
| **7.1 … multiple outcome measurements within the outcome domain?** | No | No | No | No | No | No | No | No | No | No | No | No | No | No | No | No | No | No | No | No |
| **7.2 ... multiple analyses of the intervention-outcome relationship?** | PN | PN | PN | PN | PN | PN | PN | PN | PN | PN | PN | PN | PN | PN | PN | PN | PN | PN | PN | PN |
| **7.3 ... different subgroups?** | PN | PN | PN | PN | PN | PN | PN | PN | PN | PN | PN | PN | PN | PN | PN | PN | PN | PN | PN | PN |
| **Risk of bias judgment** | Low | Low | Low | Low | Low | Low | Low | Low | Low | Low | Low | Low | Low | Low | Low | Low | Low | Low | Low | Low |
| **What is the predicted direction of bias due to confounding?** | FE | FE | FE | FE | FE | FE | FE | FE | FE | FE | FE | FE | FE | FE | FE | FE | FE | FE | FE | FE |
| **Risk of bias judgment** | Moderate | Moderate | Moderate | Moderate | Moderate | Moderate | Moderate | Moderate | Moderate | Moderate | Moderate | Moderate | Moderate | Moderate | Moderate | Moderate | Moderate | Moderate | Moderate | Moderate |
| **What is the predicted direction of bias due to confounding?** | FE | FE | FE | FE | FE | FE | FE | FE | FE | FE | FE | FE | FE | FE | FE | FE | FE | FE | FE | FE |

***Abbreviations:*** PY: Probably Yes; PN: Probably No; NI: No Information; FE: Favours experimental.

**etable 6.3 Risk of Bias in Non-randomized Studies of Interventions (ROBINS-I): 2018-2021**

| **Author** | Rada-Fernandez de | Shin | Castelló | Bahrami | Kim | Toorang | Jafari Nasab | Nojomi | Jafari Nasab | Moussa |
| --- | --- | --- | --- | --- | --- | --- | --- | --- | --- | --- |
| **Year** | 2018 | 2018 | 2018 | 2019 | 2020 | 2020 | 2020 | 2021 | 2021 | 2021 |
| **1.Bias due to confounding** |  |  |  |  |  |  |  |  |  |  |
| **1.1 Is there potential for confounding of the effect of intervention in this study?** | Yes | Yes | Yes | Yes | Yes | Yes | Yes | Yes | Yes | Yes |
| **1.2 Was the analysis based on splitting participants' follow-up time according to intervention received?** | NI | PN | NI | NI | NI | NI | NI | PN | NI | NI |
| **1.3 Were intervention discontinuations or switches likely to be related to factors that are prognostic for the outcome?** | NI | NI | NI | NI | NI | NI | NI | NI | NI | NI |
| **1.4 Did the authors use an appropriate analysis method that controlled for all the important confounding domains?** | Yes | Yes | Yes | Yes | Yes | Yes | Yes | Yes | Yes | Yes |
| **1.5 Were confounding domains that were controlled for measured validly and reliably by the variables available in this study?** | Yes | Yes | Yes | Yes | Yes | Yes | Yes | Yes | Yes | Yes |
| **1.6 Did the authors control for any post-intervention variables that could have been affected by the intervention?** | PY | Yes | PN | Yes | Yes | PY | Yes | Yes | Yes | Yes |
| **1.7 Did the authors use an appropriate analysis method that controlled for all the important confounding domains and for time-varying confounding?** | PY | Yes | PN | PY | PY | PY | PY | PY | PY | PY |
| **1.8 Were confounding domains that were controlled for measured validly and reliably by the variables available in this study?** | Yes | Yes | NI | Yes | Yes | Yes | Yes | Yes | Yes | Yes |
| **Risk of bias judgment** | Low | Low | Moderate | Low | Low | Low | Low | Low | Low | Low |
| **What is the predicted direction of bias due to confounding?** | FE | FE | FE | FE | FE | FE | FE | FE | FE | FE |
| **2.Bias in selection of participants into the study** |  |  |  |  |  |  |  |  |  |  |
| **2.1Was selection of participants into the study (or into the analysis) based on participant characteristics observed after the start of intervention?** | No | No | Yes | Yes | Yes | Yes | Yes | No | Yes | Yes |
| **2.2 Were the post-intervention variables that influenced selection likely to be associated with intervention?** | **_** | **_** | No | No | No | No | No | **_** | No | No |
| **2.3 Were the post-intervention variables that influenced selection likely to be influenced by the outcome or a cause of the outcome?** | **_** | **_** | Yes | Yes | Yes | Yes | Yes | **_** | Yes | Yes |
| **2.4 Do start of follow-up and start of intervention coincide for most participants?** | Yes | Yes | **_** | **_** | **_** | **_** | **_** | Yes | **_** | **_** |
| **2.5. Were adjustment techniques used that are likely to correct for the presence of selection bias?** | PN | PN | **_** | **_** | **_** | **_** | **_** | PN | **_** | **_** |
| **Risk of bias judgment** | Low | Low | Moderate | Moderate | Moderate | Moderate | Moderate | Low | Moderate | Moderate |
| **What is the predicted direction of bias due to confounding?** | FE | FE | FE | FE | FE | FE | FE | FE | FE | FE |
| **3.Bias in classification of interventions** |  |  |  |  |  |  |  |  |  |  |
| **3.1 Were intervention groups clearly defined?** | Yes | Yes | Yes | Yes | Yes | Yes | Yes | Yes | Yes | Yes |
| **3.2 Was the information used to define intervention groups recorded at the start of the intervention?** | Yes | Yes | No | No | No | No | No | Yes | No | No |
| **3.3 Could classification of intervention status have been affected by knowledge of the outcome or risk of the outcome?** | No | No | Yes | Yes | Yes | Yes | Yes | No | Yes | Yes |
| **Risk of bias judgment** | Low | Low | Moderate | Moderate | Moderate | Moderate | Moderate | Low | Moderate | Moderate |
| **What is the predicted direction of bias due to confounding?** | FE | FE | Towards null | Towards null | Towards null | Towards null | Towards null | FE | Towards null | Towards null |
| **4.Bias due to deviations from intended interventions** |  |  |  |  |  |  |  |  |  |  |
| **If your aim for this study is to assess the effect of: 1) assignment to intervention, answer questions 4.1 and 4.2; 2) starting and adhering to intervention, answer questions 4.3 to 4.6** | | | | | | | | | | |
| **4.1 Were there deviations from the intended intervention beyond what would be expected in usual practice?** | NI | PN | PN | PN | PN | PN | PN | NI | PN | PN |
| **4.2 Were these deviations from intended intervention unbalanced between groups and likely to have affected the outcome?** | NI | PN | PN | PN | PN | PN | PN | NI | PN | PN |
| **4.3 Were important co-interventions balanced across intervention groups?** | **_** | **_** | **_** | **_** | **_** | **_** | **_** | **_** | **_** | **_** |
| **4.4 Was the intervention implemented successfully for most participants?** | **_** | **_** | **_** | **_** | **_** | **_** | **_** | **_** | **_** | **_** |
| **4.5 Did study participants adhere to the assigned intervention regimen?** | **_** | **_** | **_** | **_** | **_** | **_** | **_** | **_** | **_** | **_** |
| **4.6 Was an appropriate analysis used to estimate the effect of starting and adhering to the intervention?** | **_** | **_** | **_** | **_** | **_** | **_** | **_** | **_** | **_** | **_** |
| **Risk of bias judgment** | NI | Low | Low | Low | Low | Low | Low | NI | Low | Low |
| **What is the predicted direction of bias due to confounding?** | Unpredictable | FE | FE | FE | FE | FE | FE | Unpredictable | FE | FE |
| **5.Bias due to missing data** |  |  |  |  |  |  |  |  |  |  |
| **5.1 Were outcome data available for all, or nearly all, participants?** | Yes | Yes | Yes | Yes | Yes | Yes | Yes | Yes | Yes | Yes |
| **5.2 Were participants excluded due to missing data on intervention status?** | Yes | Yes | NI | Yes | Yes | Yes | Yes | Yes | Yes | Yes |
| **5.3 Were participants excluded due to missing data on other variables needed for the analysis?** | Yes | Yes | NI | Yes | Yes | Yes | Yes | Yes | Yes | Yes |
| **5.4 Are the proportion of participants and reasons for missing data similar across interventions?** | Yes | Yes | NI | Yes | Yes | Yes | Yes | Yes | Yes | Yes |
| **5.5 Is there evidence that results were robust to the presence of missing data?** | No | No | No | No | No | No | No | No | No | No |
| **Risk of bias judgment** | Low | Low | Moderate | Low | Low | Low | Low | Low | Low | Low |
| **What is the predicted direction of bias due to confounding?** | FE | FE | FE | FE | FE | FE | FE | FE | FE | FE |
| **6.Bias in measurement of outcomes** |  |  |  |  |  |  |  |  |  |  |
| **6.1 Could the outcome measure have been influenced by knowledge of the intervention received?** | No | No | No | No | No | No | No | No | No | No |
| **6.2 Were outcome assessors aware of the intervention received by study participants?** | Yes | Yes | Yes | Yes | Yes | Yes | Yes | Yes | Yes | Yes |
| **6.3 Were the methods of outcome assessment comparable across intervention groups?** | Yes | Yes | Yes | Yes | Yes | Yes | Yes | Yes | Yes | Yes |
| **6.4 Were any systematic errors in measurement of the outcome related to intervention received?** | No | No | No | No | No | No | No | No | No | No |
| **Risk of bias judgment** | Moderate | Moderate | Moderate | Moderate | Moderate | Moderate | Moderate | Moderate | Moderate | Moderate |
| **What is the predicted direction of bias due to confounding?** | FE | FE | FE | FE | FE | FE | FE | FE | FE | FE |
| **7.Bias in selection of the reported result** |  |  |  |  |  |  |  |  |  |  |
| **7.1 … multiple outcome measurements within the outcome domain?** | No | No | No | No | No | No | No | No | No | No |
| **7.2 ... multiple analyses of the intervention-outcome relationship?** | PN | PN | PN | PN | PN | PN | PN | PN | PN | PN |
| **7.3 ... different subgroups?** | PN | PN | PN | PN | PN | PN | PN | PN | PN | PN |
| **Risk of bias judgment** | Low | Low | Low | Low | Low | Low | Low | Low | Low | Low |
| **What is the predicted direction of bias due to confounding?** | FE | FE | FE | FE | FE | FE | FE | FE | FE | FE |
| **Risk of bias judgment** | Moderate | Moderate | Moderate | Moderate | Moderate | Moderate | Moderate | Moderate | Moderate | Moderate |
| **What is the predicted direction of bias due to confounding?** | FE | FE | FE | FE | FE | FE | FE | FE | FE | FE |

***Abbreviations:*** PY: Probably Yes; PN: Probably No; NI: No Information; FE: Favours experimental.
